# OTC Medication Risk Literacy, Confidence, and Safety Behaviours Among Romanian Adults: A Cross-Sectional Scenario-Based Assessment

**DOI:** 10.3390/healthcare14172886

**Published:** 2026-09-07

**Authors:** Eszter-Anna Dho-Nagy, Béla Kovács, Zsolt Kovács, Francisc Boda, Boglárka Kovács-Deák, Erika-Gyöngyi Bán, Attila Brassai

**Affiliations:** 1 The Doctoral School of Medicine and Pharmacy, George Emil Palade University of Medicine, Pharmacy, Science and Technology of Târgu Mures, 540142 Târgu Mureș, Romania; eszter-anna.dho-nagy@umfst.ro (E.-A.D.-N.); bogyo131@gmail.com (B.K.-D.); 2 Department ME1/Pharmacology, Faculty of Medicine in English, George Emil Palade University of Medicine, Pharmacy, Science and Technology of Târgu Mureș, 540142 Târgu Mureș, Romania; erika.ban@umfst.ro (E.-G.B.); attila.brassai@umfst.ro (A.B.); 3 Department F1/Biochemistry and Chemistry of Environmental Factors, Faculty of Pharmacy, George Emil Palade University of Medicine, Pharmacy, Science and Technology of Târgu Mureș, 540142 Târgu Mureș, Romania; zsolt.kovacs@umfst.ro; 4 Department F1/General and Inorganic Chemistry, Faculty of Pharmacy, George Emil Palade University of Medicine, Pharmacy, Science and Technology of Târgu Mureș, 540142 Târgu Mureș, Romania; francisc.boda@umfst.ro

**Keywords:** over-the-counter medicines, medication literacy, self-medication, medication safety, pharmacovigilance

## Abstract

**Highlights:**

**What are the main findings?**

- Most respondents correctly recognized major OTC medicine-related risks, with the highest recognition observed for severe adverse reactions, pregnancy-related medicine use, persistent symptoms requiring medical evaluation, and inappropriate NSAID use.
- Important knowledge gaps persisted for antihistamine-alcohol interactions and duplicate active ingredients, while respondents with a healthcare background demonstrated higher risk-recognition and confidence scores.

**What are the implications of the main findings?**

- Targeted educational initiatives should focus on under-recognized OTC medicine risks, particularly medicine interactions, duplicate ingredients, and the safe use of medicines in the presence of comorbidities.
- Limited awareness of adverse-effect reporting highlights the need to strengthen pharmacovigilance education and promote the use of reliable information sources, including patient information leaflets.

**Abstract:**

**Background:** The widespread availability of over-the-counter (OTC) medicines supports self-care but also requires users to recognize medication-related risks and make safe decisions without direct professional supervision. Existing studies have often examined medication literacy, self-medication, or pharmacovigilance separately. To address this gap, we developed a questionnaire within the conceptual framework of OTC medication risk literacy to explore scenario-based OTC medicine risk recognition, self-assessed confidence, self-reported safety behaviours, information-seeking practices, and pharmacovigilance awareness among respondents recruited through an online survey in Romania. **Methods:** A cross-sectional observational study was conducted using an anonymous Google Forms questionnaire. Data were collected between June–July 2026. The questionnaire included scenario-based items addressing common OTC medicine risks, self-assessed confidence in medication-related decision-making, self-reported safety behaviours, information sources, chronic disease and medicine use, adverse effect experience, reporting behaviour, and pharmacovigilance awareness. Associations were evaluated using chi-square tests, effect-size measures, and multivariable regression analyses. **Results:** A total of 423 respondents completed the survey. Recognition of major medication-safety risks was high for persistent symptoms requiring medical evaluation (96.5%), severe allergic reactions (95.7%), severe cutaneous adverse effects (96.2%), OTC medicine use during pregnancy (95.7%), antibiotic misuse (89.8%), and contraindications associated with chronic disease or anticoagulant therapy (91.7–92.7%). In contrast, recognition was lower for the antihistamine–alcohol interaction scenario, for which only 53.9% selected the safest response. Confidence was highest for understanding information provided on medicine packaging and patient information leaflets, whereas lower confidence was reported for recognising unsafe medicine combinations and contraindications. Most respondents reported reading medicine packaging and following recommended dosage instructions. Doctors (69.0%), pharmacists (61.2%), and patient information leaflets (56.3%) were the most used information sources. Pharmacovigilance awareness was limited, although 45.1% of respondents agreed that they knew how to report a suspected adverse effect, 35.0% disagreed and 52.3% of eligible respondents reported having previously submitted a suspected adverse effect report. Healthcare background remained independently associated with higher recognition and confidence scores after adjustment. **Conclusions:** In this online convenience sample, respondents generally recognized major OTC medicine safety risks in structured scenarios and reported several safe-use behaviours, but practical gaps remained in interaction recognition, complex risk assessment, and pharmacovigilance awareness. The findings should be interpreted as exploratory and not as representative national estimates. Scenario-based assessment may help identify educational priorities, while pharmacist counselling, clearer information, and pharmacovigilance education remain plausible strategies that require further evaluation.

## 1. Introduction

Over-the-counter (OTC) medicines have become an integral component of modern healthcare, providing accessible and effective treatment options for a wide range of self-limiting conditions without the need for a prescription [1]. Their availability supports timely symptom relief, encourages patient autonomy and may help reduce the burden on healthcare systems when used appropriately. As self-care has become an increasingly important component of healthcare delivery worldwide, the use of OTC medicines has continued to expand across diverse populations and healthcare settings. However, the accessibility that makes these medicines highly valuable also places greater responsibility on individuals, who are expected to make informed decisions regarding medicine selection, dosing, duration of use, potential interactions, and the need to seek professional medical advice when appropriate. Consequently, ensuring the safe and responsible use of OTC medicines has become an important public health priority, requiring not only access to medicines but also the knowledge and skills necessary to use them appropriately [2].

Despite their established role in self-care, OTC medicines are not without risk. Many of the most commonly used non-prescription medicines have a narrow margin between appropriate use and clinically relevant harm when dosing recommendations are exceeded, contraindications are overlooked, or concomitant medications are not considered [3]. One of the best-known examples is paracetamol, which remains a leading cause of acute liver failure in many countries [4]. Unintentional overdoses frequently occur when patients simultaneously use multiple combination products containing the same active ingredient, often without recognizing the duplication [5]. Similarly, non-steroidal anti-inflammatory drugs (NSAIDs), which are among the most commonly used forms of self-medication [6], are associated with an increased risk of gastrointestinal bleeding, acute kidney injury, and cardiovascular events, particularly in older adults [7]. These risks are further amplified when NSAIDs are combined with anticoagulants, antiplatelet agents, corticosteroids, antihypertensive drugs, or other nephrotoxic medicines [8]. At the same time, the growing use of dietary supplements and herbal products has increased the likelihood of clinically relevant herb–drug interactions [9], many of which remain poorly recognized by the general public. Certain population groups require particular caution. During pregnancy and breastfeeding, even medicines that are readily available without a prescription should be used only after careful consideration of their expected benefits and potential risks, as safety profiles vary substantially between active substances and across different stages of pregnancy [10].

Another important concern is the tendency to delay seeking professional medical advice because symptoms are initially managed through self-medication. While many minor conditions can be safely treated with OTC medicines, prolonged or inappropriate self-treatment may mask serious underlying diseases, postpone diagnosis, and increase the risk of preventable complications. In addition, adverse effects related to OTC medicines remain substantially under-recognized and under-reported [11], limiting opportunities for pharmacovigilance systems to identify emerging safety concerns and implement appropriate risk-minimization measures.

Taken together, these findings demonstrate that OTC medicines cannot be considered universally safe simply because they are available without a prescription. Their safe use depends on informed decision-making, recognition of medication-related risks, and appropriate use within the clinical context of each individual patient. Because safe OTC medicine use depends not only on access to medicines but also on the ability to recognize, interpret, and apply medication-related information appropriately, the concept of health literacy provides an important framework for understanding why some individuals may be more vulnerable to medication-related harm than others.

Health literacy is commonly understood as the ability to access, understand, appraise, and apply health information in order to make appropriate decisions related to health and healthcare. In the context of medication use, this concept becomes more specific. Medication literacy is defined as an individual’s ability to understand and act on medication-related information [12]. Limited medication literacy may contribute to inappropriate medicine use and preventable medication-related harm. However, medication safety depends on more than reading and understanding written information. In real life, individuals often must apply medication-related knowledge to practical situations: deciding whether two medicines may be combined, recognizing duplicate active ingredients, identifying contraindications in chronic disease, judging whether an online source is reliable, or understanding when symptoms require professional medical advice. They require interpretation, context, and a certain degree of clinical reasoning.

In the present study, OTC medication risk literacy was conceptualized as an applied subdomain of medication literacy focused on recognizing clinically relevant risks during self-medication, interpreting their implications in context, and selecting appropriate safety responses. The core component of this construct was scenario-based risk recognition involving duplicate active ingredients, interactions, contraindications, prolonged symptoms requiring medical evaluation, medicine use during pregnancy, inappropriate antibiotic reuse, and recognition of serious adverse effects. Pharmacotherapy literacy has previously been defined as the capacity to obtain, evaluate, calculate, and comprehend medication-related information required for appropriate decision-making [13]. In contrast, OTC medication risk literacy is not proposed as a replacement for existing literacy frameworks but as a context-specific application of medication literacy within unsupervised OTC medicine use. Within this framework, confidence, information-seeking practices, pharmacovigilance awareness, health status, and medication exposure are considered associated domains or determinants, whereas self-reported safety behaviours represent potential behavioural manifestations rather than direct measures of literacy.

Over the past decade, increasing attention has been directed toward medication literacy, self-medication practices, and the safe use of OTC medicines across diverse populations. Previous studies have shown that higher medication literacy is associated with improved understanding of medicine information and safer medicine use [14]. However, important deficiencies remain in recognizing contraindications, clinically relevant interactions, warning signs, and other medication-related risks [14]. Furthermore, confidence in medication-related decision-making does not always correspond to objective knowledge, with some individuals overestimating and others underestimating their ability to use medicines safely [15]. Self-medication practices are increasingly influenced by digital information sources. Although healthcare professionals remain important sources of advice, many individuals rely on internet search engines, online forums, and social media when making medication-related decisions [16,17]. While digital resources have improved access to health information, they have also increased exposure to inaccurate or misleading medication-related content, making it more challenging for individuals to distinguish reliable information from misinformation [16]. Awareness of pharmacovigilance and adverse effect reporting remains limited despite continued efforts to strengthen pharmacovigilance systems world-wide [18]. Under-reporting continues to reduce opportunities for early detection of medication safety signals and limits a more accurate understanding of medication-related harm.

Although medication literacy, self-medication behaviours, information-seeking practices, confidence, and pharmacovigilance awareness have each been investigated individually, they are rarely evaluated together within a framework reflecting the complex decisions encountered during routine self-medication. Existing instruments primarily assess understanding of medication information rather than the ability to recognize, interpret, and manage clinically relevant risks in real-world OTC medicine-use situations. Consequently, there remains a need for scenario-based approaches that assess the practical application of medication-related knowledge while simultaneously examining confidence, safety behaviours, information-seeking practices, and pharmacovigilance awareness [12].

The present study aimed to evaluate scenario-based OTC medicine risk recognition, self-assessed confidence, self-reported safety behaviours, information-seeking practices, and pharmacovigilance awareness among adults in Romania within the broader conceptual framework of OTC medication risk literacy. Specifically, we examined which risks were most and least frequently recognized, how confident respondents felt when making OTC medicine-related decisions, what safety behaviours and information sources they reported, and whether these patterns differed according to healthcare background. The study further explored demographic and clinical factors associated with safer OTC medicine use. These objectives were exploratory and hypothesis-generating rather than intended to establish causal relationships or population-level prevalence estimates.

Furthermore, the study was guided by the following research questions: (1) Which OTC medicine safety scenarios are most and least frequently recognised by respondents? (2) How confident do respondents feel when interpreting OTC medicine information and identifying potentially unsafe use? (3) What safe-use behaviours and information sources do respondents report? (4) What level of pharmacovigilance awareness and previous reporting behaviour is present? (5) Do response patterns differ between respondents with and without a healthcare background? These questions were exploratory and hypothesis-generating rather than designed to establish causal effects or population-level prevalence estimates.

The study also compared these outcomes between respondents with and without a healthcare background and explored demographic and clinical factors associated with safer OTC medicine use. Rather than assessing medication knowledge alone, this study was designed to examine how individuals recognize and respond to medication-related risks commonly encountered during everyday self-medication. By integrating scenario-based risk recognition with confidence, self-medication behaviours, information-seeking practices, and pharmacovigilance awareness, we aimed to provide a more clinically meaningful assessment of OTC medicine use and identify opportunities for targeted educational interventions to promote safer self-medication practices.

## 2. Materials and Methods

### 2.1. Questionnaire Development

The instrument was designed to assess scenario-based OTC medicine risk recognition together with confidence, self-reported safety behaviours, information-seeking practices, and pharmacovigilance awareness. Although developed within the conceptual framework of OTC medication risk literacy, it should be regarded as an exploratory multidomain questionnaire rather than a fully validated measure of OTC medication risk literacy or an established latent-construct instrument. The first version included a larger number of questions covering accidental overdose, drug interactions, contraindications in chronic diseases, medicine use during pregnancy, herbal products, inappropriate antibiotic use, adverse effects, and situations requiring medical advice. After internal discussion within the research team, several questions were revised, merged, or removed to improve clarity and reduce the overall completion time. The questionnaire was then reviewed by specialists in clinical pharmacology and medication safety. Their comments mainly concerned the wording of several questions and the clarity of the clinical scenarios. The final version was prepared after incorporating these suggestions.

Before wider distribution, the questionnaire was pilot tested in a small group of volunteers representing both healthcare (physicians and pharmacists, *n* = 7) and non-healthcare backgrounds (*n* = 9). Participants completed the questionnaire and provided structured feedback regarding clarity, comprehensibility, wording, and completion time. Feedback was collected through written comments and direct discussion with members of the research team. Minor wording changes were introduced before final administration.

The questionnaire was initially drafted in English, which served as the source version during questionnaire development but was not administered to participants. The Romanian and Hungarian versions were prepared by bilingual members of the research team with healthcare expertise and reviewed against the English source version by bilingual authors to assess semantic and conceptual equivalence. Discrepancies were resolved by consensus.

#### Psychometric Assessment of the Questionnaire

*Content validity (CV)* was evaluated by a panel of 11 experts comprising physicians and pharmacists. Experts rated the relevance of 44 questionnaire items using a four-point scale ranging from “not relevant” to “highly relevant.” Ratings of “quite relevant” or “highly relevant” were classified as relevant. For each item, the item-level content validity index (I-CVI) was calculated as the proportion of experts rating the item as relevant. Scale-level content validity was assessed using the average I-CVI (S-CVI/Ave) and universal agreement (S-CVI/UA). Chance-corrected agreement was evaluated using the modified kappa statistic. An I-CVI of ≥0.78 was considered acceptable for item retention.

*Exploratory factor analysis (EFA)* was conducted for two item sets: six items assessing confidence and self-perceived knowledge regarding OTC medicine use (Q22–Q27), and eight scorable items assessing OTC medicine-use behaviours and information-related habits (Q28–Q36). The multiselect item Q34 was excluded from factor analysis because it was nominal rather than ordinal. Negatively worded items Q32 and Q33 were reverse coded so that higher scores indicated safer behaviour. Principal axis factoring was applied using complete-case data. Factorability was assessed using the Kaiser–Meyer–Olkin (KMO) measure and Bartlett’s test of sphericity. Factor retention was informed by the eigenvalue pattern and interpretability of the resulting factors. Internal consistency of the scorable questionnaire items and predefined item domains that were theoretically expected to reflect a common underlying construct was assessed using Cronbach’s alpha and standardized Cronbach’s alpha coefficients. Cronbach’s alpha values of 0.7 or higher were considered as acceptable. Nominal and multiple-response items were excluded from reliability analysis. Because only one factor was retained for Q22–Q27, rotation was unnecessary. The Q28–Q36 results represent an unrotated two-factor solution.

*Confirmatory factor analysis (CFA)* was conducted using complete data from 423 respondents. Two measurement models derived from the EFA were specified: a one-factor model comprising Q22–Q27 and a correlated two-factor model comprising Q28–Q33, Q35, and Q36. Q34 was excluded because it was a nominal multiple-response item, while Q32 and Q33 were reverse-coded so that higher scores reflected safer medicine-use behaviour. Because exact WLSMV (Weighted Least Squares Mean and Variance adjusted) estimation was performed in the computational environment, CFA was performed as a preliminary correlation-based model using a maximum-likelihood discrepancy approximation applied to the ordinal item scores. Model fit was evaluated using the chi-square statistic, comparative fit index (CFI), Tucker–Lewis index (TLI), root mean square error of approximation (RMSEA), and standardized root mean square residual (SRMR). Standardized factor loadings and residual variances were examined, and average variance extracted (AVE) and composite reliability were calculated for each latent factor.

*HTMT analysis* was used to evaluate discriminant validity for the correlated two-factor Q28–Q36 model by calculating the heterotrait–monotrait ratio of correlations between the latent factors; values below 0.85 were interpreted as supporting adequate discriminant validity.

*Preliminary assessment of measurement invariance* across respondents with and without a healthcare background was conducted for the Q22–Q27 one-factor model and the Q28–Q36 correlated two-factor model. Q34 was excluded because it was a nominal multiple-response item, and Q32–Q33 were reverse-coded so that higher scores represented safer behaviour. The first stage assessed configural invariance by fitting the same factor structure simultaneously in both groups while allowing model parameters to vary. Model fit was evaluated using χ^2^, CFI, TLI, RMSEA and SRMR. Higher levels of invariance—equality of thresholds, factor loadings and residual variances—were planned only if the configural model demonstrated adequate fit. Because exact WLSMV estimation was unavailable, the analysis used a preliminary ordinal-score correlation CFA with a maximum-likelihood discrepancy approximation.

### 2.2. Study Design

This cross-sectional observational study used an anonymous, self-administered online questionnaire to evaluate scenario-based OTC medicine risk recognition, self-assessed confidence in medication-related decision-making, self-reported safety behaviours, self-medication practices, and pharmacovigilance awareness among adults living in Romania. The study also compared respondents with a healthcare background with respondents without a healthcare background and explored factors associated with reported safer OTC medicine use. Data were collected between June–July 2026. The study was conducted in accordance with the ethical principles of the Declaration of Helsinki. Ethical approval was obtained from the Scientific Research Ethics Committee of the George Emil Palade University of Medicine, Pharmacy, Science, and Technology of Târgu Mureș, decision No. 4226/18.06.2026.

### 2.3. Participants and Recruitment

Adults aged 18 years or older living in Romania were eligible to participate in this cross-sectional online survey. The questionnaire link was distributed through university mailing lists, professional networks, social media platforms, and personal contacts in order to reach respondents with and without a healthcare background. Participation was voluntary, and no financial compensation was offered.

In accordance with STROBE and CHERRIES reporting considerations for cross-sectional internet-based surveys, the questionnaire was administered as an open, anonymous online survey created in Google Forms (Google LLC, Mountain View, CA, USA). The survey link was not restricted by individual invitation codes or passwords. Before accessing the questionnaire, respondents received written information about the purpose of the study, the voluntary nature of participation, data anonymity, and their right to discontinue before submission. Electronic informed consent was required before proceeding.

The form was configured not to collect names, e-mail addresses, telephone numbers, personal identification numbers, IP addresses, cookies, or other direct identifiers, and the option to collect respondents’ e-mail addresses was disabled. The Google Forms setting limiting responses to one submission per signed-in account was enabled; however, because no personal identifiers were collected, complete individual-level duplicate-submission detection was not possible. All questionnaire items were mandatory after consent, although conditional items were interpreted according to logical eligibility. A total of 423 complete questionnaires were submitted and included in the final analysis.

### 2.4. Questionnaire Content

The questionnaire distributed to the participants included five sections (Appendix A): (1) demographic and socioeconomic characteristics (Q1–Q9); (2) scenario-based OTC medicine risk recognition (Q10–Q21); (3) self-assessed confidence and perceived OTC medicine knowledge (Q22–Q27); (4) self-reported safety behaviours and information-seeking practices (Q28–Q36); and (5) health status, medicine exposure, adverse effect experience, reporting behaviour, and pharmacovigilance awareness (Q37–Q44). The conceptual mapping of the questionnaire is presented in Table 1.

### 2.5. Statistical Analysis

Descriptive statistics were used to summarize respondent characteristics and questionnaire responses. Categorical variables were reported as frequencies and percentages, whereas continuous variables were summarized using means, standard deviations, medians, and ranges, as appropriate. Before recruitment, Cochran’s formula was used to estimate a theoretical recruitment target of 385 respondents under assumptions of probability sampling, a 95% confidence level, and a 5% margin of error. Because the present study employed non-probability convenience sampling through voluntary online recruitment, this calculation was considered only a pragmatic recruitment target and was not interpreted as evidence of population representativeness or precision.

Associations between healthcare background and questionnaire responses were evaluated using Pearson’s chi-square tests. Expected cell frequencies were examined for all contingency tables, and Fisher’s exact test was applied when chi-square assumptions were not satisfied because of sparse cell counts. Effect sizes were quantified using Cramér’s V and interpreted as negligible (<0.10), weak (0.10–0.20), moderate (0.20–0.60), or strong (>0.60).

For the scenario-based OTC medication risk-recognition score (Q10–Q21), each correct response was assigned one point, whereas incorrect responses and “I do not know” responses received zero points, resulting in a possible score range of 0–12. For the confidence score (Q22–Q27), responses were coded on five-point ordinal scales (1–5) and summed across all six items, resulting in a possible score range of 6–30, with higher scores indicating greater self-assessed confidence and perceived OTC medication knowledge. Both scores were treated as exploratory composite measures developed for the purposes of the present study rather than as predefined validated outcomes.

To evaluate whether healthcare background remained associated with OTC medication risk recognition after accounting for potential confounding factors, multivariable linear regression was used to examine associations between healthcare background and the risk-recognition score while adjusting for age group, sex, education level, residence, chronic disease status, number of regularly used prescription medicines, and OTC medication-use frequency. An analogous multivariable linear regression model was used to examine factors associated with the confidence score. Regression coefficients (β) with 95% confidence intervals (CI) were reported. Additional exploratory analyses further separated respondents with a healthcare background into healthcare students and healthcare professionals.

For multiple-response questions, each response option was analysed as a separate binary variable (selected versus not selected) using respondents as the denominator. Consequently, percentages for multiple-response items represent the proportion of respondents selecting each option rather than the proportion of all responses provided. Conditional items were interpreted using the logically eligible subgroup as the primary denominator where appropriate.

To account for multiplicity in item-level post hoc analyses, *p*-values were adjusted using the Benjamini–Hochberg false-discovery-rate (FDR) procedure. Statistical significance was defined as *p* < 0.05, and results from post hoc analyses were interpreted primarily on the basis of FDR-adjusted *p*-values.

Questionnaire validation analyses, multivariable regression models, Benjamini–Hochberg false-discovery-rate correction, and exploratory three-group comparisons were performed in R version 4.5.3 using RStudio (Posit Software, PBC, Boston, MA, USA). All other analyses were performed using Minitab^®^ 17 (Minitab LLC, State College, PA, USA).

## 3. Results

### 3.1. Questionnaire Validation

*Content validity*. Across the 44 items and 484 expert ratings, content validity was excellent. The S-CVI/Ave was 0.996, while the S-CVI/UA was 0.955, indicating universal agreement for 42 of the 44 proposed items. All items exceeded the prespecified retention threshold, with I-CVI values ranging from 0.909 to 1.000 and a mean modified kappa of 0.996. Items Q7 and Q10 each received one “somewhat relevant” rating, resulting in an I-CVI and modified kappa of 0.909; nevertheless, both remained above the retention threshold. Section-level S-CVI/Ave values ranged from 0.990 to 1.000, supporting retention of all 44 questionnaire items.

*Exploratory factor analysis*. The six confidence and self-perceived knowledge items (Q22–Q27) were suitable for factor analysis, as indicated by a KMO value of 0.855 and a significant Bartlett’s test of sphericity, χ^2^ (15) = 1870.24, *p* < 0.001. A single factor was retained, with an eigenvalue of 3.789, accounting for 63.2% of the total variance. The second eigenvalue was 0.705, supporting a unidimensional solution. Factor loadings ranged from 0.591 to 0.842, while communalities ranged from 0.349 to 0.709. Internal consistency was assessed only for item groups that were theoretically expected to reflect a common underlying construct. Cronbach’s alpha was therefore interpreted primarily for the self-perceived knowledge (α = 0.804) and confidence domain (α = 0.881), for which exploratory factor analysis supported a unidimensional structure. For heterogeneous sections addressing self-medication behaviours, information sources, medication exposure, chronic disease status, adverse effect experience, and reporting awareness, Cronbach’s alpha was not interpreted as a measure of scale reliability, because these items were designed to capture distinct descriptive characteristics rather than manifestations of a single latent construct. Collectively, the EFA results suggested a potentially unidimensional structure regarding safe OTC medicine use. However, subsequent CFA provided only partial support for this structure, indicating that the proposed measurement model requires further refinement and independent validation before confirmatory interpretation.

The eight scorable behaviour and information-related items demonstrated acceptable factorability, with a KMO value of 0.714 and a significant Bartlett’s test of sphericity, χ^2^ (28) = 870.66, *p* < 0.001. A two-factor solution was retained. The first two eigenvalues were 2.782 and 1.563, accounting for 34.8% and 19.5% of the total variance, respectively, and 54.3% cumulatively. The first factor was primarily defined by checking medicine information, interactions, active ingredients, and recommended dosing (Q28–Q31), with absolute loadings ranging from 0.574 to 0.834. The second factor was primarily associated with potentially unsafe prolonged or excessive use and reliance on social-media information (Q32, Q33, Q35, and Q36). However, Q35 and Q36 had low absolute loadings (0.280 and 0.343) and low communalities (0.131 and 0.184), indicating weak representation by the retained factors.

*Confirmatory factor analysis*. For Q22–Q27, the one-factor CFA produced mixed fit indices: χ^2^(9) = 104.61, CFI = 0.929, TLI = 0.881, RMSEA = 0.159, and SRMR = 0.053. Standardized factor loadings ranged from 0.581 to 0.850. Although composite reliability (CR = 0.884) and average variance extracted (AVE = 0.563) supported internal consistency and convergent validity, the elevated RMSEA and subthreshold TLI indicated that the hypothesized one-factor structure was only partially supported and should be regarded as preliminary. The correlated two-factor model for Q28–Q36 also showed inadequate fit, χ^2^(19) = 109.14, CFI = 0.894, TLI = 0.844, RMSEA = 0.106, and SRMR = 0.087. The safety-checking behaviour factor demonstrated satisfactory reliability and convergent validity (loadings = 0.515–0.879, CR = 0.818, AVE = 0.538). In contrast, the risk/online-information factor performed poorly (loadings = −0.325 to 0.720, CR = 0.559, AVE = 0.268), largely because Q35 and Q36 had weak negative loadings. Thus, the Q22–Q27 construct showed stronger measurement properties, whereas the Q28–Q36 structure requires theoretical and item-level refinement before confirmatory interpretation. Discriminant validity between the two proposed Q28–Q36 factors was assessed using HTMT. The HTMT value between safety-checking behaviour and risk/online-information behaviour was 0.339 (2000-resample bootstrap 95% CI [0.220, 0.481]), which was below both the conservative 0.85 and liberal 0.90 thresholds, supporting discriminant validity between the constructs. However, this finding should be interpreted alongside the weak convergent reliability and inadequate CFA fit previously observed for the risk/online-information factor. HTMT was not applicable to Q22–Q27 because it comprised a single latent factor.

*Preliminary assessment of measurement invariance*. Neither item set demonstrated adequate configural invariance. For Q22–Q27, the unconstrained multi-group model produced χ^2^(18) = 114.48, CFI = 0.917, TLI = 0.861, RMSEA = 0.113 and SRMR = 0.064. Standardized loadings ranged from 0.586 to 0.849 among respondents with a healthcare background and from 0.544 to 0.822 among respondents without a healthcare background. For Q28–Q36, the configural model produced χ^2^(38) = 127.00, CFI = 0.897, TLI = 0.849, RMSEA = 0.075 and SRMR = 0.093. Absolute standardized loadings ranged from 0.251 to 0.905 among healthcare among respondents with a healthcare background and from 0.295 to 0.844 among respondents without a healthcare background; the estimated factor correlations were 0.267 and 0.193, respectively. Because adequate configural invariance was not demonstrated, comparisons involving these scales should be interpreted descriptively and in an exploratory manner rather than as strict latent-trait comparisons.

### 3.2. Anthropometric and Socio-Demographic Characteristics (Q1–Q9)

A total of 423 volunteer respondents completed the questionnaire. The initially calculated Cochran sample size of 385 was used only as a theoretical recruitment target under probability-sampling assumptions and was not interpreted as establishing a 5% margin of error or national representativeness. Of the 423 respondents, 263 completed the Romanian-language version and 160 completed the Hungarian-language version.

Participants were aged between 18 and 78 years (Q1), with a mean age of 32.5 years. The largest age category was 18–24 years (40.4%, *n* = 171), followed by 35–50 years (23.6%, *n* = 100) and 24–35 years (22.7%, *n* = 96). Fewer respondents were aged 50–65 years (10.9%, *n* = 46) or older than 65 years (2.4%, *n* = 10). Based on BMI (Q2/Q3), most participants were classified as normal weight (18.5–25 kg/m^2^; 52.5%, *n* = 222), whereas 22.5% (*n* = 95) had overweight, 18.2% (*n* = 77) had obesity, and 6.9% (*n* = 29) were classified as underweight. The sample was predominantly female (79.1%, *n* = 334), while 20.9% (*n* = 88) were male and one respondent preferred not to disclose gender (Q4). Considering occupational status (Q5) most participants were employed (53.2%, *n* = 225) or students (38.1%, *n* = 161), with smaller proportions reporting unemployment (5.4%, *n* = 23) or retirement (3.3%, *n* = 14). Regarding perceived financial status (Q6), 42.8% (*n* = 181) reported being able to afford necessary items and some additional expenses, while 23.9% (*n* = 101) could comfortably cover basic needs and 20.6% (*n* = 87) could cover basic needs with very little remaining. Difficulty covering basic needs was reported by 5.4% (*n* = 23), and 7.3% (*n* = 31) preferred not to answer. Educational attainment (Q7) was mainly secondary education (45.4%, *n* = 192), followed by college or university education (30.5%, *n* = 129) and master’s or doctoral degrees (21.0%, *n* = 89). The sample was nearly balanced between respondents with a healthcare background (51.3%, *n* = 217) and respondents without a healthcare background (48.7%, *n* = 206). Among respondents with a healthcare background (Q8), medical students formed the largest subgroup (45.6%, *n* = 99), followed by physicians (27.6%, *n* = 60), nurses or assistants (13.4%, *n* = 29), other healthcare workers (9.7%, *n* = 21), and pharmacists (3.7%, *n* = 8). Most respondents lived in urban areas (74.9%, *n* = 317), while 25.1% (*n* = 106) lived in rural areas (Q9). A summarizing table regarding the anthropometric and sociodemographic characteristics of the cohort is presented in Supplementary Table S1.

### 3.3. Scenario-Based Recognition of OTC Medicine-Related Risks (Q10–Q21)

Recognition of OTC medicine-related risks was generally high across most scenarios (Table 2). The highest rates of correct responses were observed for persistent symptoms requiring medical evaluation (Q16), OTC medicine use during pregnancy (Q19), severe allergic reactions (Q20), severe cutaneous adverse effects (Q21), inappropriate antibiotic use (Q17), anticoagulant-associated pain-reliever use (Q13), and daily ibuprofen use in individuals with cardiovascular or renal disease (Q12). Recognition was also high for accidental paracetamol overdose (Q11) and risks associated with herbal products and dietary supplements (Q18). The lowest level of correct recognition was observed for the antihistamine–alcohol interaction scenario (Q15), for which only 53.9% of respondents selected the safest response. This item was also associated with one of the highest levels of respondent uncertainty. Interpretation of this finding should be cautious because the scenario did not distinguish between sedating and non-sedating antihistamines. Comparisons between respondents with and without a healthcare background demonstrated statistically significant differences for most scenario-based items; however, effect sizes were generally weak. Moderate associations were observed for recognition of paracetamol duplication risks (Q11; Cramér’s V = 0.244), antihistamine–alcohol interactions (Q15; Cramér’s V = 0.328), and risks associated with herbal products and dietary supplements (Q18; Cramér’s V = 0.215). Respondent-level post hoc analyses with Benjamini–Hochberg correction further showed that respondents with a healthcare background were significantly more likely than respondents without a healthcare background to select the correct responses in several medication-risk scenarios, including duplicate paracetamol exposure (Q11), long-term analgesic use (Q12), anticoagulant-related precautions (Q13), antihistamine–alcohol interactions (Q15), inappropriate antibiotic reuse (Q17), herbal product safety (Q18), and medication use during pregnancy (Q19). Respondents without a healthcare background were also significantly more likely to select “I do not know” responses for several scenarios (Supplementary Table S2).

#### Multivariable Analysis of OTC Medication Risk-Recognition Score

An overall exploratory OTC medication risk-recognition score was calculated by summing correct responses to questions Q10–Q21 (possible range: 0–12). Correct responses received one point, whereas incorrect responses and “I do not know” responses received zero points. The mean score was 10.51 ± 2.09, with a median of 11, an interquartile range of 10–12, and an observed range of 0–12. The distribution deviated from normality according to the Shapiro–Wilk test (W = 0.716, *p* < 0.001). The multivariable model included healthcare background, age group, sex, educational attainment, place of residence, chronic disease status, prescription medicine use, and OTC medicine-use frequency. The analytical sample consisted of 422 respondents. The model explained 30.9% of the variance in the exploratory risk-recognition score (adjusted R^2^ = 27.3%; F(21,400) = 8.52, *p* < 0.001). In multivariable linear regression (Table 3), healthcare background remained significantly associated with higher recognition scores (β = 1.20, 95% CI 0.82–1.57, *p* < 0.001). Male sex was independently associated with lower recognition scores (β = −0.68, 95% CI −1.12 to −0.24, *p* = 0.003), whereas urban residence was associated with higher scores (β = 0.71, 95% CI 0.30–1.11, *p* < 0.001). Respondents who reported never or almost never using OTC medicines demonstrated lower risk-recognition scores than respondents reporting OTC medicine use less than once per month (β = −1.64, 95% CI −2.35 to −0.94, *p* < 0.001). No significant independent associations were observed for age group, education level, chronic disease status, or prescription medicine use.

### 3.4. Self-Assessed Knowledge and Confidence (Q22–Q27)

Overall, respondents reported moderate to high confidence across most medication-related tasks (Table 4). Confidence was highest for understanding information provided on medicine packaging and patient information leaflets (Q22) and for recognizing adverse effects requiring medical attention (Q25). Lower confidence was observed for recognizing potentially unsafe medicine combinations (Q23) and identifying situations in which OTC medicines may be unsafe because of comorbidities or concomitant medication use (Q24). Respondents with a healthcare background consistently reported higher levels of confidence and self-perceived knowledge than respondents without a healthcare background. Significant associations with healthcare background were observed for all confidence and knowledge items (Q22–Q27), with moderate effect sizes (Cramér’s V = 0.217–0.405). The strongest associations were observed for self-perceived knowledge regarding the safe use of OTC medicines (Q26; Cramér’s V = 0.405), confidence in recognizing unsafe medicine use in the context of comorbidities or concomitant medicines (Q24; Cramér’s V = 0.377), and confidence in recognizing inappropriate medicine combinations (Q23; Cramér’s V = 0.366). Benjamini–Hochberg-adjusted post hoc analyses (Supplementary Table S2) further demonstrated that respondents without a healthcare background were overrepresented in lower-confidence categories, whereas respondents with a healthcare background were more likely to report high confidence. Significant differences were identified for several response categories across Q22–Q25, while attitudinal items (Q26–Q27) showed a clear polarization, with healthcare background respondents more likely to agree or strongly agree with positive statements regarding their knowledge and ability to use OTC medicines safely.

When respondents with a healthcare background were further separated into healthcare students and healthcare professionals, pairwise post hoc analyses revealed few differences between these two groups. No significant differences were observed for Q22–Q26 after Benjamini–Hochberg correction, whereas a small but significant difference was identified for Q27 (adjusted *p* = 0.018). In contrast, both healthcare students and healthcare professionals differed significantly from respondents without a healthcare background across most confidence and self-perceived knowledge items (Q22–Q27), indicating that the largest differences were associated with healthcare training rather than professional experience alone (Supplementary Table S3).

#### Multivariable Analysis of Confidence in OTC Medication-Related Decision-Making

To account for potential confounding, an exploratory confidence score was calculated by summing responses to questions Q22–Q27 (possible range: 6–30). The mean confidence score was 20.65 ± 5.41, with a median of 21, an interquartile range of 17–24, and an observed range of 6–30. The distribution deviated from normality according to the Shapiro–Wilk test (W = 0.979, *p* < 0.001). The multivariable model (Table 5) included healthcare background, age group, sex, educational attainment, place of residence, chronic disease status, prescription medicine use, and OTC medicine-use frequency. The analytical sample consisted of 422 respondents. In multivariable linear regression, healthcare background remained strongly associated with higher confidence scores (β = 4.13, 95% CI 3.13–5.13, *p* < 0.001). Respondents reporting OTC medicine use several times per week had higher confidence scores than those using OTC medicines less than once per month (β = 1.77, 95% CI 0.21–3.32, *p* = 0.026), whereas respondents reporting never or almost never using OTC medicines had lower confidence scores (β = −1.73, 95% CI −3.15 to −0.31, *p* = 0.017). The model explained 26.9% of the variance in the exploratory confidence score (adjusted R^2^ = 23.1%; F(21,400) = 7.01, *p* < 0.001).

### 3.5. OTC Medicine Use Habits and Information Sources (Q28–Q36)

Overall, respondents reported generally safe OTC medicine-use practices (Table 6). Most participants indicated that they routinely consulted medicine packaging or patient information leaflets before using a new OTC product, checked for duplicate active ingredients when using more than one OTC medicine, assessed potential interactions, and followed recommended dosage instructions. Conversely, the reported use of excessive doses (Q32) or prolonged self-medication despite persistent symptoms (Q33) was uncommon. Comparisons by healthcare background showed relatively few differences in self-reported behaviours. Respondents with a healthcare background were more likely to report checking for duplicate active ingredients before taking multiple OTC medicines (Q29), which represented the strongest behavioural association observed in this section (Cramér’s V = 0.206). No significant differences were observed for reading medicine packaging information (Q28), checking for interactions (Q30), adherence to recommended doses (Q31), excessive OTC medicine use (Q32), or prolonged use despite persistent symptoms (Q33). When evaluating OTC medicine safety, respondents most commonly relied on healthcare professionals and patient information leaflets, while internet searches were also frequently used. Family members, friends, and previous personal experience were reported less often as information sources. Although social media use for OTC medicine decision-making was generally uncommon and trust in online or social-media information was low, respondent-level post hoc analyses showed that respondents without a healthcare background were significantly more likely to rely on family members or friends as a source of OTC medicine information, whereas respondents with a healthcare background were significantly more likely to consult patient information leaflets (Supplementary Table S2).

Pairwise post hoc analyses of OTC medicine safety behaviours and related practices revealed few differences among the three healthcare-status groups. Significant differences were observed only for Q29, where healthcare professionals differed from both healthcare students and respondents without a healthcare background (both adjusted *p* < 0.001). No significant differences were observed between healthcare students and respondents without a healthcare background for Q29. For all other behavioural items (Q28, Q30–Q33, Q35–Q36), no significant pairwise differences remained after Benjamini–Hochberg correction.

### 3.6. Health Status, Medication Use, Adverse Effects, and Pharmacovigilance Awareness (Q37–Q44)

Approximately one-third of respondents reported having a chronic disease requiring regular treatment or monitoring, while most reported no chronic condition (Table 7). The most frequently reported chronic conditions among eligible respondents were cardiovascular disease, allergic disorders, and asthma. Most participants did not use regular prescription medicines, although a minority reported treatment with one or more prescription drugs, including a small proportion meeting criteria for polypharmacy. OTC medicine use was common across the sample. Vitamins or dietary supplements, NSAIDs, paracetamol/acetaminophen, and cold or flu medicines were the most frequently reported OTC products. Healthcare background respondents reported somewhat greater use of NSAIDs, whereas vitamin and supplement use was similarly common in both groups. A minority of respondents reported having experienced a suspected adverse effect following OTC medicine use. Among respondents who reported a suspected adverse effect or were uncertain whether they had experienced an adverse effect, reporting behaviour was limited, with a little more than half indicating that they had reported the event to a healthcare professional or pharmacovigilance reporting system. In addition, a proportion of respondents were unaware that adverse effect reporting was possible. Self-reported knowledge regarding how and where to report suspected medicine-related adverse effects was also variable, with substantial numbers of respondents expressing uncertainty or disagreement with this statement. Overall, the findings suggest that OTC medicine use is widespread in both healthcare background and non-healthcare background respondents, whereas awareness of pharmacovigilance reporting systems and previous adverse effect reporting practices remain comparatively limited.

## 4. Discussion

The present study explored scenario-based recognition of OTC medicine-related risks, self-assessed confidence, medication-safety behaviours, information-seeking practices, and pharmacovigilance awareness among Romanian adults within an exploratory framework of OTC medication risk literacy. Rather than focusing exclusively on factual medication knowledge, the study examined respondents’ ability to recognise and respond appropriately to clinically relevant situations encountered during self-medication. Overall, participants demonstrated high recognition of several major medication-safety risks, particularly those involving pregnancy, persistent symptoms requiring medical evaluation, severe allergic reactions, severe cutaneous adverse effects, and contraindications associated with chronic diseases or concomitant medicines. However, performance was less consistent for scenarios involving medicine interactions, duplicate active ingredients, and herbal product safety, suggesting that self-medication may be more challenging when risk assessment requires contextual interpretation rather than recognition of obvious warning signs.

A central finding was the contrast between recognition of dramatic or highly visible clinical risks and recognition of less apparent medication-related hazards. Respondents correctly identified severe allergic reactions, severe cutaneous adverse effects, and circumstances requiring medical consultation at very high rates. In contrast, substantially lower recognition was observed for the antihistamine–alcohol interaction scenario, duplicate paracetamol exposure, and risks related to herbal products and dietary supplements. Similar patterns have been reported in medication-literacy and self-medication studies conducted in other populations, where respondents generally demonstrate strong recognition of acute or severe medication-related dangers but perform less well when identifying interactions, contraindications, or dosage-related risks [19,20]. These findings suggest that public medication-safety messages concerning severe warning signs have been relatively successful, whereas interaction-related and context-dependent risks remain less well understood. Consequently, educational efforts may benefit from focusing more specifically on recognising hidden risks that commonly occur during routine self-medication.

The antihistamine–alcohol interaction scenario in particular merits discussion because it generated the lowest proportion of correct responses and the highest degree of uncertainty. This finding may reflect the fact that interaction risks are inherently more complex than recognising a severe adverse effect or obvious contraindication. Interpretation of this result should nevertheless be cautious because the questionnaire did not distinguish between sedating and non-sedating antihistamines. Despite this limitation, the finding highlights a broader challenge of self-medication: individuals must frequently evaluate risks that are not immediately apparent and that require integration of information from multiple sources rather than simple recall of a single safety rule [21].

The antibiotic-reuse scenario was also included intentionally despite antibiotics not being OTC medicines. Although this may appear conceptually distinct from OTC use, the underlying decision-making process is closely related to self-medication behaviour. Individuals must decide whether to treat symptoms independently, determine whether a medicine is appropriate for the current condition, and recognise when professional assessment is required. The high proportion of correct responses in this scenario is encouraging; however, inappropriate antibiotic reuse remains a persistent international public-health concern and illustrates the broader importance of medication-related risk recognition during unsupervised medicine use [22,23].

Another important theme emerging from the study concerns the relationship between confidence and demonstrated recognition of medication-related risks. Respondents reported the greatest confidence for understanding information presented in patient information leaflets and for recognising severe adverse effects, whereas confidence was lower for identifying medicine interactions and risks associated with chronic illnesses or concomitant medication use. This pattern is consistent with previous literature showing that perceived competence and objectively demonstrated medication knowledge are related but distinct constructs. Individuals may overestimate or underestimate their ability to make safe medication-related decisions, and confidence alone cannot be considered evidence of adequate medication literacy [24]. These findings support the conceptual distinction adopted in the present study, where confidence was considered a correlate of OTC medication risk literacy rather than a direct measure of literacy itself.

The comparison between respondents with and without a healthcare background revealed statistically significant differences for several knowledge and confidence domains. Participants with a healthcare background generally showed higher recognition of medication-related risks and reported greater confidence in medication-related decision-making. However, effect sizes were predominantly weak to moderate, indicating that healthcare training explains only part of the variability in OTC medicine safety. Equally important, several findings demonstrated substantial similarities between groups. Recognition of severe allergic reactions, severe cutaneous adverse effects, pregnancy-related caution, and persistent symptoms requiring professional evaluation was consistently high regardless of healthcare background. These similarities suggest that some medication-safety messages are well established within the wider population and that future educational interventions may be more effective when focused on complex interaction-related risks rather than universally recognised warning signs [20,21]. Although the majority of subgroup analyses were exploratory and based on item-level comparisons, an additional multivariable analysis demonstrated that healthcare background remained independently associated with higher OTC medication risk-recognition scores after adjustment for demographic characteristics, residence, chronic disease status, prescription medicine use, and OTC medicine-use frequency. This suggests that the observed differences cannot be explained solely by differences in demographic composition between groups. Nevertheless, residual confounding remains possible, and the cross-sectional design precludes causal interpretation.

Notably, healthcare students and healthcare professionals demonstrated remarkably similar performance in both scenario-based recognition and confidence-related items. This finding suggests that many medication-safety competencies may develop during healthcare education before entry into independent professional practice.

The behavioural findings provide additional insight into the practical application of medication-related knowledge. Most respondents reported reading patient information leaflets, checking active ingredients, monitoring potential interactions, and following recommended dosage instructions. These behaviours are encouraging because they represent practical actions that may reduce preventable medication-related harm. At the same time, a meaningful minority reported inconsistent engagement with these practices. Similar discrepancies between knowledge and behaviour have been described in previous self-medication studies, suggesting that recognition of medication-related risks does not always translate directly into safer medicine-use practices. This observation further supports the view that medication safety is influenced by a combination of knowledge, confidence, behavioural habits, and contextual factors rather than by knowledge alone [24,25].

Information-seeking patterns also revealed an interesting contrast. Respondents frequently reported consulting healthcare professionals and patient information leaflets when evaluating OTC medicine safety, and trust in social-media information was generally low. Nevertheless, awareness of pharmacovigilance reporting systems and actual adverse effect reporting behaviour were considerably less developed. This discrepancy suggests that although respondents actively seek information before using medicines, they are less familiar with their potential role in post-marketing medicine-safety surveillance. Similar deficiencies in public awareness of adverse effect reporting have been reported internationally and remain a persistent challenge for pharmacovigilance systems [26,27].

The observed gaps in pharmacovigilance awareness have broader implications for contemporary medication-safety policy. Recent policy frameworks increasingly promote the integration of predictive analytics, advanced signal-detection methods, and data-driven surveillance approaches alongside traditional spontaneous reporting systems [28,29]. Although these technologies can improve the identification of emerging medication-safety signals, their effectiveness continues to depend on the availability of timely and accurate reports from medicine users. Thus, public engagement in adverse effect reporting remains essential. Furthermore, evidence from vulnerable populations has demonstrated that multimorbidity, polypharmacy, age, and complex medication regimens substantially increase susceptibility to adverse effects [30,31]. Although the present study focused on OTC medicines, these findings are directly relevant to self-medicating individuals with chronic diseases or concomitant prescription medicines. Contemporary policy recommendations therefore increasingly advocate integrated medication-safety strategies combining public education, pharmacist involvement, standardized care pathways, multidisciplinary collaboration, and strengthened pharmacovigilance systems [32,33]. Such approaches may provide a useful framework for improving OTC medicine safety beyond educational interventions alone.

The pattern of information-seeking behaviour further highlights the central role of healthcare professionals in OTC medicine safety. Doctors and pharmacists were the most frequently reported sources used when deciding whether an OTC medicine was safe, followed by patient information leaflet. Post hoc analyses suggested differences in the type of supplementary information sources used. Respondents with a healthcare background were more likely to report consulting patient information leaflets, whereas respondents without a healthcare background more frequently relied on family members or friends. This pattern may reflect differences in familiarity with medicine-related information resources and risk communication. This reliance on professional and regulated sources is reassuring and supports the continued role of pharmacists as accessible medication-safety educators. At the same time, nearly half of respondents reported using internet searches, whereas social media use was comparatively uncommon and trust in online or social media information was low. These findings suggest that respondents may distinguish between general online searching and social media-based advice; however, the frequent use of internet sources still warrants attention. The quality of online health information is variable, and the recent literature emphasizes that social media and influencer-driven health advice may be affected by limited expertise, commercial incentives, incomplete discussion of harms, and inconsistent credibility standards [34,35]. Thus, digital health literacy should be incorporated into OTC medicine safety education, with emphasis on identifying reliable sources and verifying online information with pharmacists or physicians.

Taken together, the findings provide preliminary support for the usefulness of examining OTC medicine safety as a multidimensional phenomenon encompassing risk recognition, confidence, medication-use behaviours, information-seeking practices, and pharmacovigilance awareness. Importantly, the psychometric analyses should be interpreted cautiously. Content validity was excellent, and exploratory analyses identified interpretable structures for the confidence-related and behaviour-related domains. However, confirmatory factor analyses demonstrated only partial support for the proposed models, and the preliminary assessment of measurement invariance across respondents with and without a healthcare background was not established. Consequently, the questionnaire should be regarded as an exploratory multidomain instrument rather than a fully validated questionnaire developed within the conceptual framework of OTC medication risk literacy. Similarly, the risk-recognition score and confidence score should be interpreted as exploratory composite measures derived from observed questionnaire responses rather than as predefined validated outcomes. Because adequate measurement invariance across healthcare background groups was not demonstrated, comparisons involving the confidence score should be interpreted as exploratory comparisons of summed observed responses rather than comparisons of an established invariant latent construct. The present findings provide preliminary evidence that the proposed framework may be useful for organizing related domains of OTC medicine use, while also highlighting the need for further instrument refinement and validation.

Several limitations should be considered when interpreting the findings. The cross-sectional design precludes causal inference, and the study relied on a self-selected online convenience sample that was predominantly young, female, urban, highly educated, and connected to university or professional networks, thereby limiting generalisability to the broader Romanian population. Individuals at potentially greater risk of medication-related harm, including older adults, those with multimorbidity, polypharmacy, lower health literacy, or reduced digital access, may therefore be underrepresented. In addition, the absence of a measurable response rate limits assessment of participation bias. Self-reported behaviours may also have been affected by recall bias and social desirability bias, particularly because many questions concerned recommended medication-safety practices. Although bilingual review procedures were applied, language-equivalence limitations may persist because formal cross-cultural adaptation and back-translation were not performed. Furthermore, despite the application of false-discovery-rate correction, the large number of statistical comparisons increases the possibility that some findings occurred by chance. Several scenario-based items demonstrated ceiling effects, reducing their ability to discriminate between respondents with higher levels of medication-related knowledge. Finally, responses to hypothetical clinical scenarios may not accurately reflect actual medication-use decisions or behaviours in real-world settings.

## 5. Conclusions

This study provides exploratory evidence regarding OTC medication risk literacy, confidence, safety behaviours, information-seeking practices, and pharmacovigilance awareness among adults in Romania. Overall, respondents demonstrated good recognition of several major OTC medicine safety risks, particularly situations involving persistent symptoms, pregnancy, antibiotic misuse, severe allergic reactions, severe cutaneous adverse effects, and OTC medicine use in the context of chronic disease or anticoagulant therapy. However, important gaps remained in the recognition of more subtle but common risk situations, especially the antihistamine–alcohol interaction, duplicate active ingredients, and the potential risks of herbal products and dietary supplements. Confidence was also uneven, with participants feeling more secure when interpreting written medicine information or recognizing serious adverse effects, but less confident when assessing interactions, contraindications, or risk in the presence of comorbidities and concomitant medication use.

The findings suggest that safe self-medication requires more than access to OTC products and written instructions. It depends on practical risk literacy: the ability to apply medication-related information to real-life decisions, recognize when professional advice is needed, avoid unsafe combinations or duplicated active ingredients, and report suspected adverse effects. Although healthcare background was associated with higher knowledge and confidence in several domains, the generally weak to moderate effect sizes indicate that medication safety education should target both the general public and healthcare-related groups. Pharmacists and physicians remain central sources of trusted advice, while the frequent use of internet searches highlights the need for accessible, accurate, and user-friendly digital medication safety resources.

In conclusion, the results support the value of scenario-based approaches for identifying practical gaps in OTC medicine safety knowledge and self-care decision-making. Targeted educational interventions should focus on commonly overlooked risks, including alcohol-related interactions, duplicate active ingredients, supplement-related interactions, and situations requiring professional consultation. Strengthening pharmacist-supported counselling, improving public pharmacovigilance awareness, and developing reliable digital information tools may help translate OTC medicine availability into safer and more informed self-medication practices.

## Figures and Tables

**Table 1:** Conceptual mapping of questionnaire domains, response formats, and analytical approaches.

Items	Domain	Format	Analysis Approach and Rationale
Q1–Q9	Demographic, socioeconomic, educational, residential, and healthcare background variables.	Categorical and numerical responses	Described the sample and supported subgroup comparisons by healthcare background.
Q10–Q21	Scenario-based OTC medicine risk recognition	Multiple-choice scenarios with one safest response	Correct/incorrect coding assessed recognition of key OTC risks, including duplication, interactions, contraindications, pregnancy-related caution, persistent symptoms, antibiotic misuse, and serious adverse effects.
Q22–Q27	Self-assessed confidence and perceived OTC medicine knowledge	Five-point Likert-type scales	Ordinal responses were analysed descriptively and by healthcare background; internal consistency and factor analyses were applied to this item set.
Q28–Q36	Self-reported safety behaviour and information-seeking practices	Frequency, agreement, and multiple-response items	Items captured patient information leaflet use, checking active ingredients and interactions, dose adherence, prolonged or excessive use, professional advice, internet use, and social-media trust.
Q37–Q44	Health status, medicine exposure, adverse effect experience, reporting behaviour, and pharmacovigilance awareness	Categorical, yes/no, frequency, and awareness items	Descriptive and subgroup analyses provided clinical context for OTC exposure and pharmacovigilance-related awareness.

**Table 2:** Distribution of correct responses to OTC medicine safety and risk scenarios among respondents with and without a healthcare background.

Item	Question/Scenario	Correct Response	Overall *n* (%)	HB, *n*/N (%)	NHB, *n*/N (%)	χ^2^ Test	Cramér’s V	Effect Size
Q10	Which statement is the most accurate?	Over-the-counter medicines can cause side effects or interactions if not used properly	355 (83.9%)	198/217 (91.2%)	157/206 (76.2%)	χ^2^ (1, 423) = 16.600 ***p* < 0.0001**	0.198	W
Q11	Someone takes paracetamol for a headache and also takes a cold medicine that also contains paracetamol. What is the main risk?	The person may accidentally take too much paracetamol	369 (87.2%)	207/217 (95.4%)	162/206 (78.6%)	χ^2^ (1, 423) = 25.144 ***p* < 0.0001**	**0.244**	**M**
Q12	A person with heart or kidney disease wants to take ibuprofen daily for pain. What is the safest action or solution?	Ask a doctor or pharmacist before daily use	392 (92.7%)	208/217 (95.9%)	184/206 (89.3%)	χ^2^ (1, 423) = 5.715 ***p* = 0.017**	0.116	W
Q13	A person taking a blood thinner wants to use an over-the-counter pain reliever. What is the safest action?	Ask a healthcare professional before use	388 (91.7%)	208/217 (95.9%)	180/206 (87.4%)	χ^2^ (1, 423) = 8.913 ***p* = 0.003**	0.145	W
Q14	A person with poorly controlled high blood pressure wants to take a nasal decongestant. What should they do?	Ask a pharmacist or doctor before use	346 (81.8%)	194/217 (89.4%)	152/206 (73.8%)	χ^2^ (1, 423) = 16.272 ***p* < 0.0001**	0.196	W
Q15	Someone takes an antihistamine for allergy symptoms and wants to drink alcohol. What is the main safety concern?	Alcohol may increase drowsiness or dizziness	228 (53.9%)	152/217 (70.0%)	76/206 (36.9%)	χ^2^ (1, 423) = 45.421 ***p* < 0.0001**	**0.328**	**M**
Q16	Someone has used over-the-counter medicines for several weeks, but the symptoms still persist. What is the safest action?	Consult a doctor for evaluation	408 (96.5%)	215/217 (99.1%)	193/206 (93.7%)	χ^2^ (1, 423) = 7.467 ***p* = 0.006**	0.133	W
Q17	Someone has cold symptoms with a runny nose and sore throat and wants to take an antibiotic left over from a previous prescription. What is the safest advice?	Viral infections generally do not require an antibiotic, and leftover antibiotics should not be used without medical advice	380 (89.8%)	207/217 (95.4%)	173/206 (84.0%)	χ^2^ (1, 423) = 13.845 ***p* = 0.0002**	0.181	W
Q18	Which statement is the most accurate?	Herbal products and dietary supplements can also cause side effects or interactions	364 (86.1%)	203/217 (93.5%)	161/206 (78.2%)	χ^2^ (1, 423) = 19.60 ***p* < 0.0001**	**0.215**	**M**
Q19	A pregnant person wants to take an over-the-counter medicine for pain, cold symptoms, or allergy. What is the safest approach?	Ask a pharmacist, doctor, or midwife before use	405 (95.7%)	214/217 (98.6%)	191/206 (92.7%)	χ^2^ (1, 423) = 7.636 ***p* = 0.006**	0.134	W
Q20	After taking an over-the-counter medicine, someone develops swelling of the lips or throat and difficulty breathing. What should they do?	Seek emergency medical help immediately	405 (95.7%)	210/217 (96.8%)	195/206 (94.7%)	χ^2^ (1, 423) = 0.698 ***p* = 0.403**	0.041	N
Q21	After starting a new over-the-counter medicine, someone develops a widespread rash with fever, blisters, peeling skin, or sores in or around the mouth or eyes. What should they do?	Stop the suspected medicine and seek urgent medical advice	407 (96.2%)	211/217 (97.2%)	196/206 (95.1%)	χ^2^ (1, 423) = 0.759 ***p* = 0.384**	0.042	N

Abbreviations: HB = healthcare background (N = 217); NHB = non-healthcare background (N = 206). Percentages are within group. Effect size labels are W = weak, M = moderate, N = negligible. Statistically significant results are highlighted with bold.

**Table 3:** Multivariable model of OTC medication risk recognition among survey respondents. β = unstandardized regression coefficient; CI = confidence interval.

Variable	β	95% CI	*p*
Healthcare background	1.20	0.82 to 1.57	***<0.001***
Age 18–24 y	0.98	−0.27 to 2.24	*0.125*
Age 24–35 y	0.85	−0.46 to 2.15	*0.208*
Age 35–50 y	0.66	−0.61 to 1.92	*0.308*
Age 50–65 y	−0.15	−1.44 to 1.13	*0.815*
Male sex	−0.68	−1.12 to −0.24	***0.003***
Master’s degree/PhD	0.07	−0.43 to 0.58	*0.778*
Primary school	−0.17	−1.28 to 0.94	*0.761*
Secondary school	−0.16	−0.67 to 0.35	*0.537*
Urban residence	0.71	0.30 to 1.11	***<0.001***
Chronic disease, unsure	−0.61	−1.51 to 0.28	*0.181*
Chronic disease, prefer not to answer	−0.71	−2.36 to 0.94	*0.398*
Chronic disease, yes	0.28	−0.22 to 0.78	*0.272*
Prescription medicine: 1	−0.04	−0.58 to 0.50	*0.880*
Prescription medicine: 2–4	−0.47	−1.12 to 0.18	*0.159*
Prescription medicine: 5+	−0.95	−2.48 to 0.58	*0.223*
Prescription medicine: prefer not to answer	−2.92	−4.35 to −1.49	***<0.001***
OTC use 1–3/month	−0.18	−0.80 to 0.44	*0.568*
OTC use less than monthly	−0.29	−0.87 to 0.30	*0.337*
OTC use never/almost never	−1.64	−2.35 to −0.94	***<0.001***
OTC use once weekly	−0.86	−1.82 to 0.11	*0.082*

Abbreviations: Model statistics: *n* = 422; R^2^ = 0.309; adjusted R^2^ = 0.273; residual standard error = 1.79; F(21,400) = 8.52; *p* < 0.001. Reference categories were: non-healthcare background, age ≥ 65 years, female sex, bachelor’s degree, rural residence, no chronic disease, no prescription medicine use, and OTC medicine use less than once per month. Statistically significant results are highlighted with bold.

**Table 4:** Self-assessed confidence and perceived knowledge regarding safe over-the-counter medicine use among respondents with and without a healthcare background.

Item	Question/Statement	Response Category	Overall *n* (%)	HB, *n*/N (%)	NHB, *n*/N (%)	χ^2^ Test	Cramér’s V	Effect Size
Q22	How confident do you feel in understanding the instructions on over-the-counter medicine packaging or patient information leaflets?	Not confident at all	18 (4.3%)	4/217 (1.8%)	14/206 (6.8%)	χ^2^ (4, 423) = 33.535 ***p* < *0.0001***	**0.282**	**M**
		Slightly confident	21 (5.0%)	5/217 (2.3%)	16/206 (7.8%)			
		Moderately confident	127 (30.0%)	55/217 (25.3%)	72/206 (35.0%)			
		Quite confident	72 (17.0%)	31/217 (14.3%)	41/206 (19.9%)			
		Very confident	185 (43.7%)	122/217 (56.2%)	63/206 (30.6%)			
Q23	How confident do you feel in recognizing which two medicines should not be taken together?	Not confident at all	42 (9.9%)	10/217 (4.6%)	32/206 (15.5%)	χ^2^ (4, 423) = 56.573 ***p* < *0.0001***	**0.366**	**M**
		Slightly confident	89 (21.0%)	24/217 (11.1%)	65/206 (31.6%)			
		Moderately confident	165 (39.0%)	92/217 (42.4%)	73/206 (35.4%)			
		Quite confident	40 (9.5%)	27/217 (12.4%)	13/206 (6.3%)			
		Very confident	87 (20.6%)	64/217 (29.5%)	23/206 (11.2%)			
Q24	How confident do you feel in recognizing when an over-the-counter medicine may be unsafe because of another illness or a regularly taken medicine?	Not confident at all	44 (10.4%)	8/217 (3.7%)	36/206 (17.5%)	χ^2^ (4, 423) = 60.095 ***p* < *0.0001***	**0.377**	**M**
		Slightly confident	86 (20.3%)	26/217 (12.0%)	60/206 (29.1%)			
		Moderately confident	157 (37.1%)	84/217 (38.7%)	73/206 (35.4%)			
		Quite confident	42 (9.9%)	30/217 (13.8%)	12/206 (5.8%)			
		Very confident	94 (22.2%)	69/217 (31.8%)	25/206 (12.1%)			
Q25	How confident do you feel in recognizing when a side effect or skin reaction after taking a medicine requires medical help?	Not confident at all	12 (2.8%)	1/217 (0.5%)	11/206 (5.3%)	χ^2^ (4, 423) = 19.903 ***p* < *0.0001***	**0.217**	**M**
		Slightly confident	20 (4.7%)	7/217 (3.2%)	13/206 (6.3%)			
		Moderately confident	90 (21.3%)	38/217 (17.5%)	52/206 (25.2%)			
		Quite confident	64 (15.1%)	31/217 (14.3%)	33/206 (16.0%)			
		Very confident	237 (56.0%)	140/217 (64.5%)	97/206 (47.1%)			
Q26	I have adequate knowledge about the safe use of over-the-counter medicines.	Strongly disagree	22 (5.2%)	4/217 (1.8%)	18/206 (8.7%)	χ^2^ (4, 423) = 69.524 ***p* < *0.0001***	**0.405**	**M**
		Disagree	67 (15.8%)	16/217 (7.4%)	51/206 (24.8%)			
		Neutral	128 (30.3%)	53/217 (24.4%)	75/206 (36.4%)			
		Agree	166 (39.2%)	109/217 (50.2%)	57/206 (27.7%)			
		Strongly agree	40 (9.5%)	35/217 (16.1%)	5/206 (2.4%)			
Q27	I think I know enough about over-the-counter medicines to use most of them safely without consulting a professional.	Strongly disagree	32 (7.6%)	4/217 (1.8%)	28/206 (13.6%)	χ^2^ (4, 423) = 57.992 ***p* < *0.0001***	**0.370**	**M**
		Disagree	109 (25.8%)	41/217 (18.9%)	68/206 (33.0%)			
		Neutral	128 (30.3%)	62/217 (28.6%)	66/206 (32.0%)			
		Agree	124 (29.3%)	83/217 (38.2%)	41/206 (19.9%)			
		Strongly agree	30 (7.1%)	27/217 (12.4%)	3/206 (1.5%)			

Abbreviations: HB = healthcare background (N = 217); NHB = non-healthcare background (N = 206). Percentages are within group. Effect size labels are W = weak, M = moderate, and N = negligible. Statistically significant results are highlighted with bold.

**Table 5:** Multivariable linear regression analysis of factors associated with confidence in OTC medication-related decision-making (Q22–Q27).

Predictor	β Coefficient	95% CI	*p*-Value
Healthcare background	4.13	3.13 to 5.13	***<0.001***
Age 18–24 y	1.35	−1.98 to 4.69	*0.426*
Age 24–35 y	2.34	−1.13 to 5.81	*0.186*
Age 35–50 y	1.62	−1.74 to 4.98	*0.344*
Age 50–65 y	0.25	−3.16 to 3.67	*0.885*
Male sex	−0.82	−1.99 to 0.36	*0.173*
Master’s degree/PhD	0.24	−1.11 to 1.58	*0.730*
Primary school	−0.15	−3.10 to 2.79	*0.918*
Secondary school	−0.03	−1.38 to 1.33	*0.967*
Urban residence	1.36	0.28 to 2.45	***0.014***
Chronic disease, unsure	−2.43	−4.81 to −0.05	***0.046***
Chronic disease, prefer not to answer	−5.16	−9.55 to −0.78	***0.021***
Chronic disease, yes	0.27	−1.06 to 1.59	*0.694*
Prescription medicine: 1	−0.56	−1.99 to 0.87	*0.442*
Prescription medicine: 2–4	0.68	−1.06 to 2.42	*0.444*
Prescription medicine: 5+	−0.30	−4.37 to 3.77	*0.884*
Prescription medicine: prefer not to answer	−1.36	−5.16 to 2.44	*0.483*
OTC use several times per week	1.77	0.21 to 3.32	***0.026***
OTC use 1–3/month	0.59	−0.57 to 1.76	*0.317*
OTC use never/almost never	−1.73	−3.15 to −0.31	***0.017***
OTC use once weekly	0.71	−1.60 to 3.03	*0.545*

Abbreviations: Reference categories were: non-healthcare background, age ≥ 65 years, female sex, bachelor’s degree, rural residence, no chronic disease, no prescription medicine use, and OTC medicine use less than once per month. Statistically significant results are highlighted with bold.

**Table 6:** Self-reported OTC medicine safety behaviours, information-seeking practices, and trust in digital sources.

Item	Question/Statement	Response Category	Overall *n* (%)	HB, *n*/N (%)	NHB, *n*/N (%)	χ^2^ Test	Cramér’s V	Effect Size
Q28	Before taking a new over-the-counter medicine, I usually read the medicine packaging or patient information leaflet.	Never	9 (2.1%)	4/217 (1.8%)	5/206 (2.4%)	χ^2^ (4, 423) = 2.667 *p* = 0.615	0.079	N
		Rarely	34 (8.0%)	18/217 (8.3%)	16/206 (7.8%)			
		Sometimes	55 (13.0%)	32/217 (14.7%)	23/206 (11.2%)			
		Always	209 (49.4%)	100/217 (46.1%)	109/206 (52.9%)			
		Frequently	116 (27.4%)	63/217 (29.0%)	53/206 (25.7%)			
Q29	Before I start taking two over-the-counter medicines during the same period, I usually check whether they contain the same active ingredient.	Never	22 (5.2%)	4/217 (1.8%)	18/206 (8.7%)	χ^2^ (4, 423) = 17.899, ***p* = 0.001**	**0.206**	**M**
		Rarely	29 (6.9%)	11/217 (5.1%)	18/206 (8.7%)			
		Sometimes	57 (13.5%)	30/217 (13.8%)	27/206 (13.1%)			
		Always	220 (52.0%)	129/217 (59.4%)	91/206 (44.2%)			
		Frequently	95 (22.5%)	43/217 (19.8%)	52/206 (25.2%)			
Q30	Before using an over-the-counter medicine, I usually check whether it may interact with other medicines or products.	Never	21 (5.0%)	5/217 (2.3%)	16/206 (7.8%)	χ^2^ (4, 423) = 8.475 *p* = 0.076	0.142	W
		Rarely	32 (7.6%)	18/217 (8.3%)	14/206 (6.8%)			
		Sometimes	60 (14.2%)	27/217 (12.4%)	33/206 (16.0%)			
		Always	199 (47.0%)	108/217 (49.8%)	91/206 (44.2%)			
		Frequently	111 (26.2%)	59/217 (27.2%)	52/206 (25.2%)			
Q31	When using over-the-counter medicines, I usually follow the recommended dosage and dosing intervals on the medicine packaging or patient information leaflet.	Never	3 (0.7%)	1/217 (0.5%)	2/206 (1.0%)	χ^2^ (4, 423) = 5.495 *p* = 0.240	0.114	W
		Rarely	11 (2.6%)	4/217 (1.8%)	7/206 (3.4%)			
		Sometimes	20 (4.7%)	11/217 (5.1%)	9/206 (4.4%)			
		Always	284 (67.1%)	138/217 (63.6%)	146/206 (70.9%)			
		Frequently	105 (24.8%)	63/217 (29.0%)	42/206 (20.4%)			
Q32	If symptoms are severe or persistent, I sometimes take an over-the-counter medicine more often or at a higher dose than recommended.	Never	222 (52.5%)	115/217 (53.0%)	107/206 (51.9%)	χ^2^ (4, 423) = 6.048 *p* = 0.196	0.120	W
		Rarely	114 (27.0%)	58/217 (26.7%)	56/206 (27.2%)			
		Sometimes	59 (13.9%)	27/217 (12.4%)	32/206 (15.5%)			
		Always	11 (2.6%)	4/217 (1.8%)	7/206 (3.4%)			
		Frequently	17 (4.0%)	13/217 (6.0%)	4/206 (1.9%)			
Q33	I continue using an over-the-counter medicine for a long time if symptoms persist.	Never	217 (51.3%)	113/217 (52.1%)	104/206 (50.5%)	χ^2^ (4, 423) = 3.189 *p* = 0.527	0.087	N
		Rarely	124 (29.3%)	66/217 (30.4%)	58/206 (28.2%)			
		Sometimes	60 (14.2%)	25/217 (11.5%)	35/206 (17.0%)			
		Always	6 (1.4%)	4/217 (1.8%)	2/206 (1.0%)			
		Frequently	16 (3.8%)	9/217 (4.1%)	7/206 (3.4%)			
Q34 ^a)^	Which source do you most often use when deciding whether an over-the-counter medicine is safe?	Pharmacist	259 (61.2%)	122/217 (56.2%)	137/206 (66.5%)	–	–	–
		Doctor	292 (69.0%)	151/217 (69.6%)	141/206 (68.4%)			
		Patient information leaflet	238 (56.3%)	139/217 (64.1%)	99/206 (48.1%)			
		Information on the medicine packaging	44 (10.4%)	19/217 (8.8%)	25/206 (12.1%)			
		Internet search	201 (47.5%)	104/217 (47.9%)	97/206 (47.1%)			
		Family or friends	67 (15.8%)	21/217 (9.7%)	46/206 (22.3%)			
		Previous personal experience	106 (25.1%)	57/217 (26.3%)	49/206 (23.8%)			
		Not verifying	2 (0.5%)	0/217 (0.0%)	2/206 (1.0%)			
		Other	4 (0.9%)	4/217 (1.8%)	0/206 (0.0%)			
Q35	How often do you use social media platforms—such as TikTok, Instagram, YouTube, or Facebook—to decide whether an over-the-counter medicine is safe?	Never	282 (66.7%)	150/217 (69.1%)	132/206 (64.1%)	χ^2^ (4, 423) = 4.638 *p* = 0.326	0.105	W
		Rarely	73 (17.3%)	35/217 (16.1%)	38/206 (18.4%)			
		Sometimes	48 (11.3%)	20/217 (9.2%)	28/206 (13.6%)			
		Always	1 (0.2%)	0/217 (0.0%)	1/206 (0.5%)			
		Frequently	19 (4.5%)	12/217 (5.5%)	7/206 (3.4%)			
Q36	I trust information about over-the-counter medicines found online or on social media as much as information from healthcare professionals.	Strongly disagree	220 (52.0%)	116/217 (53.5%)	104/206 (50.5%)	χ^2^ (4, 423) = 3.911 *p* = 0.418	0.096	N
		Disagree	119 (28.1%)	58/217 (26.7%)	61/206 (29.6%)			
		Neutral	69 (16.3%)	33/217 (15.2%)	36/206 (17.5%)			
		Agree	12 (2.8%)	7/217 (3.2%)	5/206 (2.4%)			
		Strongly agree	3 (0.7%)	3/217 (1.4%)	0/206 (0.0%)			

Abbreviations: HB = healthcare background (N = 217); NHB = non-healthcare background (N = 206). Percentages are within group. Effect size labels are W = weak, M = moderate, and N = negligible. Statistically significant results are highlighted with bold.

**Table 7:** Health status, medication use, OTC medicine exposure, adverse effect experience, and reporting behaviour by healthcare status.

Item	Question/Statement	Response Category	Overall *n* (%)	HB, *n*/N (%)	NHB, *n*/N (%)
Q37	Do you currently have any chronic disease that requires regular treatment or monitoring?	Yes	125 (29.6%)	68/217 (31.3%)	57/206 (27.7%)
		No	275 (65.0%)	137/217 (63.1%)	138/206 (67.0%)
		I am not sure	17 (4.0%)	10/217 (4.6%)	7/206 (3.4%)
		I prefer not to answer	6 (1.4%)	2/217 (0.9%)	4/206 (1.9%)
Q38	If yes, what chronic disease(s) do you have?	Cardiovascular disease	35/125 (28.0%)	11/68 (16.2%)	24/57 (42.1%)
		Previous thrombosis/blood clot	5/125 (4.0%)	3/68 (4.4%)	2/57 (3.5%)
		Kidney disease	1/125 (0.8%)	1/68 (1.5%)	0/57 (0.0%)
		Liver disease	3/125 (2.4%)	3/68 (4.4%)	0/57 (0.0%)
		Diabetes	4/125 (3.2%)	2/68 (2.9%)	2/57 (3.5%)
		Gastric ulcer/reflux disease	6/125 (4.8%)	3/68 (4.4%)	3/57 (5.3%)
		Asthma	17/125 (13.6%)	13/68 (19.1%)	4/57 (7.0%)
		Allergic disease	29/125 (23.2%)	19/68 (27.9%)	10/57 (17.5%)
		Previous drug allergy or severe skin reaction	8/125 (6.4%)	6/68 (8.8%)	2/57 (3.5%)
		Other	62/125 (49.6%)	38/68 (55.9%)	24/57 (42.1%)
		I prefer not to answer	2/125 (1.6%)	0/68 (0.0%)	2/57 (3.5%)
		Not applicable	298 (70.4%)	149/217 (68.6%)	149/206 (72.3%)
Q39	How many prescription medicines do you currently take regularly?	None	286 (67.6%)	145/217 (66.8%)	141/206 (68.4%)
		1	75 (17.7%)	44/217 (20.3%)	31/206 (15.0%)
		2–4	48 (11.3%)	23/217 (10.6%)	25/206 (12.1%)
		5 or more	6 (1.4%)	0/217 (0.0%)	6/206 (2.9%)
		I prefer not to answer	8 (1.9%)	5/217 (2.3%)	3/206 (1.5%)
Q40	How often do you use over-the-counter medicines?	Never or almost never	62 (14.7%)	32/217 (14.7%)	30/206 (14.6%)
		Less than once a month	183 (43.3%)	90/217 (41.5%)	93/206 (45.1%)
		1–3 times per month	108 (25.5%)	57/217 (26.3%)	51/206 (24.8%)
		Once a week	19 (4.5%)	14/217 (6.5%)	5/206 (2.4%)
		Several times per week	51 (12.1%)	24/217 (11.1%)	27/206 (13.1%)
Q41	Which over-the-counter medicines do you use most often?	NSAID	242 (57.2%)	136/217 (62.7%)	106/206 (51.5%)
		Paracetamol/acetaminophen	205 (48.5%)	114/217 (52.5%)	91/206 (44.2%)
		Cold or flu medicines	163 (38.5%)	79/217 (36.4%)	84/206 (40.8%)
		Nasal sprays	112 (26.5%)	66/217 (30.4%)	46/206 (22.3%)
		Antacids or reflux medicines	51 (12.1%)	30/217 (13.8%)	21/206 (10.2%)
		Allergy medicines	71 (16.8%)	41/217 (18.9%)	30/206 (14.6%)
		Vitamins or dietary supplements	246 (58.2%)	123/217 (56.7%)	123/206 (59.7%)
		Herbal/Natural products	85 (20.1%)	51/217 (23.5%)	34/206 (16.5%)
		Sleep aids	12 (2.8%)	8/217 (3.7%)	4/206 (1.9%)
		Other	22 (5.2%)	10/217 (4.6%)	12/206 (5.8%)
		I do not use OTC medicines	26 (6.1%)	9/217 (4.1%)	17/206 (8.3%)
Q42	Have you ever experienced a side effect or adverse effect after using an over-the-counter medicine?	Yes	65 (15.4%)	38/217 (17.5%)	27/206 (13.1%)
		No	319 (75.4%)	156/217 (71.9%)	163/206 (79.1%)
		I am not sure	39 (9.2%)	23/217 (10.6%)	16/206 (7.8%)
Q43	Have you ever reported a suspected side effect related to a medicine to a healthcare professional or official reporting system?	Yes	34/65 (52.3%)	18/38 (47.3%)	16/27 (59.3%)
		No	21/65 (32.3%)	15/38 (39.4%)	6/27 (22.2%)
		I did not know this was possible	9/65 (13.8%)	4/38 (10.5%)	5/27 (18.5%)
		I am not sure	1/65 (1.5%)	1/38 (2.6%)	0/27 (0.0%)
		Not applicable	358 (84.6%)	179/217 (82.4%)	179/206 (86.9%)
Q44	I know where or how to report a suspected side effect related to a medicine.	Strongly disagree	69 (16.3%)	19/217 (8.8%)	50/206 (24.3%)
		Disagree	79 (18.7%)	42/217 (19.4%)	37/206 (18.0%)
		Neutral/neither disagree nor agree	84 (19.9%)	39/217 (18.0%)	45/206 (21.8%)
		Agree	127 (30.0%)	77/217 (35.5%)	50/206 (24.3%)
		Strongly agree	64 (15.1%)	40/217 (18.4%)	24/206 (11.7%)

Abbreviations: HB = healthcare background (N = 217); NHB = non-healthcare background (N = 206). Percentages are within group.

## Data Availability

The data presented in this study are available upon request from the corresponding author due to privacy or ethical restrictions.

## Supplementary Materials

The following supporting information can be downloaded at https://www.mdpi.com/article/10.3390/healthcare14172886/s1, Supplementary Table S1: Anthropometric and sociodemographic characteristics of the participants. Supplementary Table S2: Respondent Choices and Post-Hoc Analysis of Over-the-Counter Medicine Perceptions. Supplementary Table S3: Pairwise post hoc comparisons for scenario-based recognition of OTC medicine-related risks (Q10–Q21) and self-assessed knowledge and confidence items (Q22–Q27) by healthcare status.

## Abbreviations

The following abbreviations are used in this manuscript:

AVE	Average Variance Extracted
BH	Benjamini–Hochberg
BMI	Body Mass Index
CHERRIES	Checklist for Endorsing Reporting Guidelines for Internet E-Surveys
CFA	Confirmatory Factor Analysis
CFI	Comparative Fit Index
CI	Confidence Interval
CR	Composite Reliability
CVI	Content Validity Index
EFA	Exploratory Factor Analysis
FDR	False Discovery Rate
HB	Healthcare Background
HP	Healthcare Professional
HS	Healthcare Student
HTMT	Heterotrait–Monotrait Ratio of Correlations
I-CVI	Item-Level Content Validity Index
KMO	Kaiser–Meyer–Olkin Measure
NHB	Non-Healthcare Background
NSAID	Non-Steroidal Anti-Inflammatory Drug
OTC	Over the Counter
Q	Questionnaire Item
RMSEA	Root Mean Square Error of Approximation
S-CVI/Ave	Scale-Level Content Validity Index Based on Average Agreement
S-CVI/UA	Scale-Level Content Validity Index Based on Universal Agreement
SRMR	Standardized Root Mean Square Residual
STROBE	Strengthening the Reporting of Observational Studies in Epidemiology
TLI	Tucker–Lewis Index
WLSMV	Weighted Least Squares Mean and Variance Adjusted

## Appendix A

**General information**
  **Over-the-counter medicines and their safe use**
  *Dear Participant,*
  *This questionnaire explores your knowledge, experiences, and habits related to the safe use of over-the-counter (non-prescription) medicines.*
  *The questionnaire is anonymous, participation is voluntary, and completion takes about 8–10 min. The collected data will be used exclusively for scientific research purposes.*
  *By continuing with the questionnaire, you consent to take part in the study.*
  *Thank you for your help!*
  ***(Q1) Please state your age: years:*** _______
  ***(Q2) Body weight***: _____ kg
  ***(Q3) Height***: ______ cm
  ***Part I—Anthropometric and socio-demographic characteristics***
  ***(Q4) Gender (Choose one answer)***

(a) Female
(b) Male
(c) Prefer not to say

***(Q5) Employment status (Choose one answer)***

(a) Student
(b) Employed
(c) Unemployed
(d) Retired

***(Q6) How would you describe your current financial situation? (Choose one answer)***

(a) I can afford most necessary things and some extra expenses
(b) I can comfortably cover basic needs
(c) I can cover basic needs, but have very little left over
(d) I have difficulty covering basic needs
(e) I prefer not to answer

***(Q7) What is the highest level of education you have completed? (Choose one answer)***

(a) Primary school
(b) Secondary school
(c) College or university degree
(d) Master’s degree/PhD

***(Q8) Which of the following categories best describes you? (Choose one answer)***

(a) General population, not a healthcare worker
(b) Doctor
(c) Pharmacist
(d) Nurse/assistant
(e) Other healthcare professional
(f) Medical student

***(Q9) Which of the following best describes the area where you currently live? (Choose one answer)***

(a) Urban area/town or city
(b) Rural area/village

***Part II—Knowledge of OTC medicine-related risks***
  *For each question, please select the answer you think is most appropriate. If you are unsure, choose the “I don’t know” option.*
  **(Q10) *Which statement is most accurate? (Choose one answer)***

(a) Over-the-counter medicines are always safe because they do not require a prescription
(b) Over-the-counter medicines can cause side effects or interactions if not used properly
(c) Over-the-counter medicines cannot cause serious harm
(d) Over-the-counter medicines cannot interact with other prescription medicines
(e) I don’t know

***(Q11) Someone takes paracetamol for a headache and also uses a cold remedy that also contains paracetamol. What is the main risk? (Choose one answer)***

(a) The person may accidentally take too much paracetamol
(b) The medicines cancel each other’s effect
(c) The combination is always safe because both medicines are OTC
(d) The cold medicine becomes ineffective
(e) I don’t know

***(Q12) A person with heart or kidney disease wants to take ibuprofen daily for pain. What is the safest action or solution? (Choose one answer)***

(a) Ask a doctor or pharmacist before daily use
(b) Automatically combine it with another pain reliever
(c) Daily ibuprofen is always safe because it is OTC
(d) I don’t know

***(Q13) A person taking a blood-thinning medicine wants to use an over-the-counter painkiller. What is the safest action? (Choose one answer)***

(a) Ask a healthcare professional before use
(b) Take both medicines together without any concern
(c) Temporarily stop the blood thinner
(d) They can use it freely because it is OTC
(e) I don’t know

***(Q14) A person with poorly controlled high blood pressure wants to take a decongestant for nasal congestion. What should they do? (Choose one answer)***

(a) Ask a pharmacist or doctor before use
(b) They can use it freely because it is OTC
(c) They can use the lowest dose without asking for advice
(d) I don’t know

***(Q15) Someone takes an antihistamine for allergic symptoms and would like to drink alcohol. What is the main safety concern? (Choose one answer)***

(a) Alcohol may increase drowsiness or dizziness
(b) Alcohol prevents allergic reactions
(c) The antihistamine becomes completely ineffective
(d) The combination is safer if taken with food
(e) I don’t know

***(Q16) Someone has been using over-the-counter medicines for several weeks, but their symptoms persist. What is the safest action? (Choose one answer)***

(a) Continue indefinitely without medical advice
(b) See a doctor for further evaluation
(c) Permanently increase the dose
(d) Combine several over-the-counter medicines
(e) I don’t know

***(Q17) A person has cold symptoms with runny nose and sore throat and wants to take leftover antibiotics from a previous prescription. What is the safest advice? (Choose one answer)***

(a) Viral infections generally do not require an antibiotic, and leftover antibiotics should not be used without medical advice
(b) For a cold, antibiotics should always be started as soon as possible
(c) Automatically combine the antibiotic with any OTC cold medicine
(d) Double the antibiotic dose to recover more quickly
(e) I don’t know

***(Q18) Which statement is most accurate? (Choose one answer)***

(a) Herbal products and dietary supplements can also cause side effects or interactions
(b) Dietary supplements cannot interact with prescription medicines
(c) Herbal products are always safer than conventional medicines
(d) Natural products never affect bleeding risk or liver or kidney function
(e) I don’t know

***(Q19) A pregnant person wants to take an over-the-counter medicine for pain, cold symptoms, or allergy. What is the safest approach? (Choose one answer)***

(a) Use any over-the-counter medicine because it is not prescription-only
(b) Ask a pharmacist, doctor, or midwife before use
(c) Take a lower dose without asking anyone for advice
(d) Use only a herbal product, because these are always safe
(e) I don’t know

***(Q20) After taking an over-the-counter medicine, someone develops swelling of the lips or throat and has difficulty breathing. What should they do? (Choose one answer)***

(a) Seek emergency medical help immediately
(b) Take another OTC medicine and rest
(c) Take the same medicine again later
(d) Wait to see if it improves
(e) I don’t know

***(Q21) After starting a new over-the-counter medicine, someone develops widespread skin rash with fever, blisters, peeling skin, and sores in the mouth or around the eyes. What should they do? (Choose one answer)***

(a) Continue the medicine and wait one week
(b) Stop the suspected medicine and urgently seek medical advice
(c) Apply cosmetic cream and ignore it
(d) Take a higher dose the next day
(e) I don’t know

***Part III—Self-assessed knowledge and confidence***
  ***(Q22) How confident do you feel in understanding the instructions on the medicine packaging or patient information leaflet of over-the-counter medicines? (Choose one answer)***

(a) Not confident at all
(b) Slightly confident
(c) Moderately confident
(d) Quite confident
(e) Very confident

***(Q23) How confident do you feel in recognizing which two medicines should not be taken together? (Choose one answer)***

(a) Not confident at all
(b) Slightly confident
(c) Moderately confident
(d) Quite confident
(e) Very confident

***(Q24) How confident do you feel in recognizing when an over-the-counter medicine might be unsafe because of another illness or a regularly taken medicine? (Choose one answer)***

(a) Not confident at all
(b) Slightly confident
(c) Moderately confident
(d) Quite confident
(e) Very confident

***(Q25) How confident do you feel in recognizing when a side effect or skin reaction after taking a medicine requires medical attention? (Choose one answer)***

(a) Not confident at all
(b) Slightly confident
(c) Moderately confident
(d) Quite confident
(e) Very confident

***(Q26) I have adequate knowledge about the safe use of over-the-counter medicines. (Choose one answer)***

(a) Strongly disagree
(b) Disagree
(c) Neutral/neither agree nor disagree
(d) Agree
(e) Strongly agree

***(Q27) I think I know enough about over-the-counter medicines to use most of them safely without consulting a professional***. (Choose one answer)

(a) Strongly disagree
(b) Disagree
(c) Neutral/neither agree nor disagree
(d) Agree
(e) Strongly agree

***Part IV—OTC medicine use habits and information sources***
  ***(Q28) Before taking a new over-the-counter medicine, I usually read the medicine packaging or the patient information leaflet. (Choose one answer)***

(a) Never
(b) Rarely
(c) Sometimes
(d) Always
(e) Frequently

***(Q29) Before taking two over-the-counter medicines during the same period, I usually check whether they contain the same active ingredient. (Choose one answer)***

(a) Never
(b) Rarely
(c) Sometimes
(d) Always
(e) Frequently

***(Q30) Before using an over-the-counter medicine, I usually check whether it can interact with other medicines or products I am taking. (Choose one answer)***

(a) Never
(b) Rarely
(c) Sometimes
(d) Always
(e) Frequently

***(Q31) When using over-the-counter medicines, I usually follow the recommended dose and dosing intervals stated on the medicine packaging or in the patient information leaflet. (Choose one answer)***

(a) Never
(b) Rarely
(c) Sometimes
(d) Always
(e) Frequently

***(Q32) If symptoms are severe or persistent, I sometimes take over-the-counter medicines more often or in higher doses than recommended. (Choose one answer)***

(a) Never
(b) Rarely
(c) Sometimes
(d) Always
(e) Frequently

***(Q33) I continue using an over-the-counter medicine for a long time if my symptoms persist. (Choose one answer)***

(a) Never
(b) Rarely
(c) Sometimes
(d) Always
(e) Frequently

***(Q34) Which source do you use most often when deciding whether an over-the-counter medicine is safe? (Select all that apply)***

(a) Pharmacist
(b) Doctor
(c) Patient information leaflet
(d) Information on the medicine packaging
(e) Internet search
(f) Family or friends
(g) Previous personal experience
(h) Not verifying
(i) Other

***(Q35) How often do you use social media platforms—such as TikTok, Instagram, YouTube, or Facebook—to decide whether an over-the-counter medicine is safe? (Choose one answer)***

(a) Never
(b) Rarely
(c) Sometimes
(d) Always
(e) Frequently

***(Q36) I trust information about over-the-counter medicines found online or on social media just as much as information from healthcare professionals. (Choose one answer)***

(a) Strongly disagree
(b) Disagree
(c) Neutral/neither agree nor disagree
(d) Agree
(e) Strongly agree

***Part V—Health status, medication use, adverse effects, and reliability analysis***
  ***(Q37) Do you currently have any chronic illness that requires regular treatment or monitoring? (Choose one answer)***

(a) Yes
(b) No
(c) I am not sure
(d) I prefer not to answer

***(Q38) If yes, which chronic condition(s) do you have? (Select all that apply)***

(a) Cardiovascular disease, including high blood pressure
(b) Previous thrombosis/blood clot
(c) Kidney disease
(d) Liver disease
(e) Diabetes
(f) Peptic ulcer/reflux disease
(g) Asthma/chronic obstructive pulmonary disease
(h) Allergic disease
(i) Previous drug allergy or severe skin reaction
(j) Other
(k) I prefer not to answer

***(Q39) How many prescribed (prescription) medicines do you currently take regularly? (Choose one answer)***

(a) None
(b) 1
(c) 2–4
(d) 5 or more
(e) I prefer not to answer

***(Q40) How often do you use over-the-counter medicines? (Choose one answer)***

(a) Never or almost never
(b) Less often than once a month
(c) 1–3 times per month
(d) Once a week
(e) Several times per week

***(Q41) Which over-the-counter medicines do you use most frequently? (Select all that apply)***

(a) Non-steroidal anti-inflammatory painkillers, such as ibuprofen or naproxen
(b) Paracetamol/acetaminophen
(c) Cold or flu remedies
(d) Nasal sprays
(e) Antacids or reflux medicines
(f) Allergy medicines
(g) Vitamins or dietary supplements
(h) Herbal preparations
(i) Sleep aids
(j) Other
(k) I do not use over-the-counter medicines

***(Q42) Have you ever experienced a side effect or undesirable reaction after using an over-the-counter medicine? (Choose one answer)***

(a) Yes
(b) No
(c) I am not sure

***(Q43) Have you ever reported a suspected side effect related to a medicine to a healthcare professional or an official reporting system? (Choose one answer.)***

(a) Yes
(b) No
(c) I did not know this was possible
(d) I am not sure

***(Q44) I know where or how to report a suspected side effect related to a medicine. (Choose one answer)***

(a) Strongly disagree
(b) Disagree
(c) Neutral/neither agree nor disagree
(d) Agree
(e) Strongly agree
