# Supplementary material for: OTC Medication Risk Literacy, Confidence, and Safety Behaviours Among Romanian Adults: A Cross-Sectional Scenario-Based Assessment
Source: Healthcare (Basel). 2026 Sep 7;14(17):2886. doi: 10.3390/healthcare14172886 (PMC13565273; doi:10.3390/healthcare14172886)
Supplement: Supplementary file 1 [file healthcare-14-02886-s001.zip › healthcare-4495687-Supplementary_Table_S2.pdf]

**Supplementary Table S2.** Respondent Choices and Post-Hoc Analysis of Over-the-Counter Medicine Perceptions. Legend: HB – respondents with healthcare background, NHB – respondents with non-healthcare background, BH - Benjamini-Hochberg adjusted *p* for False Discovery Rate. Statistically significant results are shaded with green.

| Question | Response item | HB | NHB | Raw *p*-value | BH adjusted *p*-value | Significance |
|---|---|---|---|---|---|---|
| Q10 | Over-the-counter medicines are always safe because they do not require a prescription | 4/217 (1.8%) | 11/206 (5.3%) | 0.066 | 0.147 | No |
| | Over-the-counter medicines can cause side effects or interactions if not used properly | 198/217 (91.2%) | 157/206 (76.2%) | <0.001 | <0.001 | Yes |
| | Over-the-counter medicines cannot cause serious harm | 2/217 (0.9%) | 10/206 (4.9%) | 0.018 | 0.051 | No |
| | Over-the-counter medicines cannot interact with other prescription medicines | 6/217 (2.8%) | 2/206 (1.0%) | 0.286 | 0.449 | No |
| | I don't know | 7/217 (3.2%) | 26/206 (12.6%) | <0.001 | 0.002 | Yes |
| Q11 | The person may accidentally take too much paracetamol | 207/217 (95.4%) | 162/206 (78.6%) | <0.001 | <0.001 | Yes |
| | The medicines cancel each other's effect | 2/217 (0.9%) | 7/206 (3.4%) | 0.098 | 0.212 | No |
| | The combination is always safe because both medicines are OTC | 2/217 (0.9%) | 4/206 (1.9%) | 0.439 | 0.610 | No |
| | The cold medicine becomes ineffective | 1/217 (0.5%) | 6/206 (2.9%) | 0.062 | 0.141 | No |
| | I don't know | 5/217 (2.3%) | 27/206 (13.1%) | <0.001 | <0.001 | Yes |
| Q12 | Ask a doctor or pharmacist before daily use | 208/217 (95.9%) | 184/206 (89.3%) | 0.014 | 0.042 | Yes |
| | Automatically combine it with another pain reliever | 2/217 (0.9%) | 1/206 (0.5%) | 1.000 | 1.000 | No |
| | Daily ibuprofen is always safe because it is OTC | 3/217 (1.4%) | 3/206 (1.5%) | 1.000 | 1.000 | No |
| | I don't know | 4/217 (1.8%) | 18/206 (8.7%) | 0.002 | 0.007 | Yes |
| Q13 | Ask a healthcare professional before use | 208/217 (95.9%) | 180/206 (87.4%) | 0.002 | 0.009 | Yes |
| | Take both medicines together without any concern | 3/217 (1.4%) | 4/206 (1.9%) | 0.718 | 0.824 | No |
| | Temporarily stop the blood thinner | 0/217 (0.0%) | 3/206 (1.5%) | 0.115 | 0.240 | No |
| | They can use it freely because it is OTC | 3/217 (1.4%) | 4/206 (1.9%) | 0.718 | 0.824 | No |
| | I don't know | 3/217 (1.4%) | 15/206 (7.3%) | 0.003 | 0.011 | Yes |

*– continues on the next page –*

– continues from the previous page –

| Question | Response item | HB | NHB | Raw *p*-value | BH adjusted *p*-value | Significance |
|---|---|---|---|---|---|---|
| Q14 | Ask a pharmacist or doctor before use | 194/217 (89.4%) | 152/206 (73.8%) | <0.001 | <0.001 | Yes |
| | They can use it freely because it is OTC | 6/217 (2.8%) | 8/206 (3.9%) | 0.593 | 0.744 | No |
| | They can use the lowest dose without asking for advice | 14/217 (6.5%) | 23/206 (11.2%) | 0.120 | 0.246 | No |
| | I don’t know | 3/217 (1.4%) | 23/206 (11.2%) | <0.001 | <0.001 | Yes |
| Q15 | Alcohol may increase drowsiness or dizziness | 152/217 (70.0%) | 76/206 (36.9%) | <0.001 | <0.001 | Yes |
| | Alcohol prevents allergic reactions | 2/217 (0.9%) | 2/206 (1.0%) | 1.000 | 1.000 | No |
| | The antihistamine becomes completely ineffective | 44/217 (20.3%) | 62/206 (30.1%) | 0.025 | 0.068 | No |
| | The combination is safer if taken with food | 0/217 (0.0%) | 4/206 (1.9%) | 0.055 | 0.133 | No |
| | I don’t know | 19/217 (8.8%) | 62/206 (30.1%) | <0.001 | <0.001 | Yes |
| Q16 | See a doctor for further evaluation | 215/217 (99.1%) | 193/206 (93.7%) | 0.003 | 0.011 | Yes |
| | Continue indefinitely without medical advice | 0/217 (0.0%) | 2/206 (1.0%) | 0.237 | 0.395 | No |
| | I don’t know | 2/217 (0.9%) | 11/206 (5.3%) | 0.010 | 0.032 | Yes |
| Q17 | Viral infections generally do not require an antibiotic, and leftover antibiotics should not be used without medical advice | 207/217 (95.4%) | 173/206 (84.0%) | <0.001 | <0.001 | Yes |
| | For a cold, antibiotics should always be started as soon as possible | 3/217 (1.4%) | 8/206 (3.9%) | 0.132 | 0.256 | No |
| | Automatically combine the antibiotic with any OTC cold medicine | 1/217 (0.5%) | 6/206 (2.9%) | 0.062 | 0.141 | No |
| | I don’t know | 6/217 (2.8%) | 19/206 (9.2%) | 0.006 | 0.023 | Yes |

– continues on the next page –

– continues from the previous page –

| Question | Response item | HB | NHB | Raw *p*-value | BH adjusted *p*-value | Significance |
|---|---|---|---|---|---|---|
| Q18 | Herbal products and dietary supplements can also cause side effects or interactions | 203/217 (93.5%) | 161/206 (78.2%) | <0.001 | <0.001 | Yes |
| | Dietary supplements cannot interact with prescription medicines | 2/217 (0.9%) | 7/206 (3.4%) | 0.098 | 0.212 | No |
| | Herbal products are always safer than conventional medicines | 4/217 (1.8%) | 15/206 (7.3%) | 0.009 | 0.029 | Yes |
| | Natural products never affect bleeding risk or liver or kidney function | 1/217 (0.5%) | 4/206 (1.9%) | 0.205 | 0.356 | No |
| | I don't know | 7/217 (3.2%) | 19/206 (9.2%) | 0.014 | 0.042 | Yes |
| Q19 | Ask a pharmacist, doctor, or midwife before use | 214/217 (98.6%) | 191/206 (92.7%) | 0.003 | 0.011 | Yes |
| | Take a lower dose without asking anyone for advice | 0/217 (0.0%) | 3/206 (1.5%) | 0.115 | 0.240 | No |
| | Use only a herbal product, because these are always safe | 0/217 (0.0%) | 1/206 (0.5%) | 0.487 | 0.656 | No |
| | I don't know | 3/217 (1.4%) | 11/206 (5.3%) | 0.029 | 0.077 | No |
| Q20 | Seek emergency medical help immediately | 210/217 (96.8%) | 195/206 (94.7%) | 0.339 | 0.503 | No |
| | Take another OTC medicine and rest | 1/217 (0.5%) | 1/206 (0.5%) | 1.000 | 1.000 | No |
| | Take the same medicine again later | 1/217 (0.5%) | 0/206 (0.0%) | 1.000 | 1.000 | No |
| | Wait to see if it improves | 1/217 (0.5%) | 2/206 (1.0%) | 0.615 | 0.744 | No |
| | I don't know | 4/217 (1.8%) | 8/206 (3.9%) | 0.250 | 0.407 | No |
| Q21 | Stop the suspected medicine and seek urgent medical advice | 211/217 (97.2%) | 196/206 (95.1%) | 0.313 | 0.475 | No |
| | Continue the medicine and wait a week | 1/217 (0.5%) | 0/206 (0.0%) | 1.000 | 1.000 | No |
| | Take a higher dose the next day | 0/217 (0.0%) | 2/206 (1.0%) | 0.237 | 0.395 | No |
| | I don't know | 5/217 (2.3%) | 8/206 (3.9%) | 0.406 | 0.571 | No |

– continues on the next page –

– continues from the previous page –

| Question | Response item | HB | NHB | Raw *p*-value | BH adjusted *p*-value | Significance |
|---|---|---|---|---|---|---|
| Q22 | Not confident at all | 4/217 (1.8%) | 14/206 (6.8%) | 0.015 | 0.042 | Yes |
| | Slightly confident | 5/217 (2.3%) | 16/206 (7.8%) | 0.013 | 0.038 | Yes |
| | Moderately confident | 55/217 (25.3%) | 72/206 (35.0%) | 0.034 | 0.090 | No |
| | Quite confident | 31/217 (14.3%) | 41/206 (19.9%) | 0.154 | 0.295 | No |
| | Very confident | 122/217 (56.2%) | 63/206 (30.6%) | <0.001 | <0.001 | Yes |
| Q23 | Not confident at all | 10/217 (4.6%) | 32/206 (15.5%) | <0.001 | <0.001 | Yes |
| | Slightly confident | 24/217 (11.1%) | 65/206 (31.6%) | <0.001 | <0.001 | Yes |
| | Moderately confident | 92/217 (42.4%) | 73/206 (35.4%) | 0.163 | 0.307 | No |
| | Quite confident | 27/217 (12.4%) | 13/206 (6.3%) | 0.045 | 0.112 | No |
| | Very confident | 64/217 (29.5%) | 23/206 (11.2%) | <0.001 | <0.001 | Yes |
| Q24 | Not confident at all | 8/217 (3.7%) | 36/206 (17.5%) | <0.001 | <0.001 | Yes |
| | Slightly confident | 26/217 (12.0%) | 60/206 (29.1%) | <0.001 | <0.001 | Yes |
| | Moderately confident | 84/217 (38.7%) | 73/206 (35.4%) | 0.546 | 0.713 | No |
| | Quite confident | 30/217 (13.8%) | 12/206 (5.8%) | 0.009 | 0.029 | Yes |
| | Very confident | 69/217 (31.8%) | 25/206 (12.1%) | <0.001 | <0.001 | Yes |
| Q25 | Not confident at all | 1/217 (0.5%) | 11/206 (5.3%) | 0.002 | 0.010 | Yes |
| | Slightly confident | 7/217 (3.2%) | 13/206 (6.3%) | 0.170 | 0.312 | No |
| | Moderately confident | 38/217 (17.5%) | 52/206 (25.2%) | 0.058 | 0.136 | No |
| | Quite confident | 31/217 (14.3%) | 33/206 (16.0%) | 0.684 | 0.801 | No |
| | Very confident | 140/217 (64.5%) | 97/206 (47.1%) | <0.001 | 0.002 | Yes |
| Q26 | Strongly disagree | 4/217 (1.8%) | 18/206 (8.7%) | 0.002 | 0.007 | Yes |
| | Disagree | 16/217 (7.4%) | 51/206 (24.8%) | <0.001 | <0.001 | Yes |
| | Neutral / neither disagree nor agree | 53/217 (24.4%) | 75/206 (36.4%) | 0.008 | 0.028 | Yes |
| | Agree | 109/217 (50.2%) | 57/206 (27.7%) | <0.001 | <0.001 | Yes |
| | Strongly agree | 35/217 (16.1%) | 5/206 (2.4%) | <0.001 | <0.001 | Yes |

– continues on the next page –

– continues from the previous page –

| Question | Response item | HB | NHB | Raw *p*-value | BH adjusted *p*-value | Significance |
|---|---|---|---|---|---|---|
| Q27 | Strongly disagree | 4/217 (1.8%) | 28/206 (13.6%) | <0.001 | <0.001 | Yes |
| | Disagree | 41/217 (18.9%) | 68/206 (33.0%) | 0.001 | 0.006 | Yes |
| | Neutral / neither disagree nor agree | 62/217 (28.6%) | 66/206 (32.0%) | 0.460 | 0.632 | No |
| | Agree | 83/217 (38.2%) | 41/206 (19.9%) | <0.001 | <0.001 | Yes |
| | Strongly agree | 27/217 (12.4%) | 3/206 (1.5%) | <0.001 | <0.001 | Yes |
| Q28 | Never | 4/217 (1.8%) | 5/206 (2.4%) | 0.746 | 0.849 | No |
| | Rarely | 18/217 (8.3%) | 16/206 (7.8%) | 0.860 | 0.923 | No |
| | Sometimes | 32/217 (14.7%) | 23/206 (11.2%) | 0.312 | 0.475 | No |
| | Always | 100/217 (46.1%) | 109/206 (52.9%) | 0.174 | 0.314 | No |
| | Frequently | 63/217 (29.0%) | 53/206 (25.7%) | 0.513 | 0.684 | No |
| Q29 | Never | 4/217 (1.8%) | 18/206 (8.7%) | 0.002 | 0.007 | Yes |
| | Rarely | 11/217 (5.1%) | 18/206 (8.7%) | 0.177 | 0.317 | No |
| | Sometimes | 30/217 (13.8%) | 27/206 (13.1%) | 0.887 | 0.944 | No |
| | Always | 129/217 (59.4%) | 91/206 (44.2%) | 0.002 | 0.008 | Yes |
| | Frequently | 43/217 (19.8%) | 52/206 (25.2%) | 0.201 | 0.353 | No |
| Q30 | Never | 5/217 (2.3%) | 16/206 (7.8%) | 0.013 | 0.038 | Yes |
| | Rarely | 18/217 (8.3%) | 14/206 (6.8%) | 0.586 | 0.744 | No |
| | Sometimes | 27/217 (12.4%) | 33/206 (16.0%) | 0.330 | 0.495 | No |
| | Always | 108/217 (49.8%) | 91/206 (44.2%) | 0.284 | 0.449 | No |
| | Frequently | 59/217 (27.2%) | 52/206 (25.2%) | 0.660 | 0.792 | No |
| Q31 | Never | 1/217 (0.5%) | 2/206 (1.0%) | 0.615 | 0.744 | No |
| | Rarely | 4/217 (1.8%) | 7/206 (3.4%) | 0.371 | 0.532 | No |
| | Sometimes | 11/217 (5.1%) | 9/206 (4.4%) | 0.821 | 0.903 | No |
| | Always | 138/217 (63.6%) | 146/206 (70.9%) | 0.121 | 0.246 | No |
| | Frequently | 63/217 (29.0%) | 42/206 (20.4%) | 0.043 | 0.109 | No |

– continues on the next page –

– continues from the previous page –

| Question | Response item | HB | NHB | Raw *p*-value | BH adjusted *p*-value | Significance |
|---|---|---|---|---|---|---|
| Q32 | Never | 115/217 (53.0%) | 107/206 (51.9%) | 0.846 | 0.915 | No |
| | Rarely | 58/217 (26.7%) | 56/206 (27.2%) | 1.000 | 1.000 | No |
| | Sometimes | 27/217 (12.4%) | 32/206 (15.5%) | 0.401 | 0.569 | No |
| | Always | 4/217 (1.8%) | 7/206 (3.4%) | 0.371 | 0.532 | No |
| | Frequently | 13/217 (6.0%) | 4/206 (1.9%) | 0.046 | 0.112 | No |
| Q33 | Never | 113/217 (52.1%) | 104/206 (50.5%) | 0.771 | 0.864 | No |
| | Rarely | 66/217 (30.4%) | 58/206 (28.2%) | 0.669 | 0.796 | No |
| | Sometimes | 25/217 (11.5%) | 35/206 (17.0%) | 0.125 | 0.247 | No |
| | Always | 4/217 (1.8%) | 2/206 (1.0%) | 0.686 | 0.801 | No |
| | Frequently | 9/217 (4.1%) | 7/206 (3.4%) | 0.801 | 0.888 | No |
| Q34 | Pharmacist | 122/217 (56.2%) | 137/206 (66.5%) | 0.036 | 0.093 | No |
| | Doctor | 151/217 (69.6%) | 141/206 (68.4%) | 0.834 | 0.910 | No |
| | Patient information leaflet | 139/217 (64.1%) | 99/206 (48.1%) | 0.001 | 0.006 | Yes |
| | Information on the medicine packaging | 19/217 (8.8%) | 25/206 (12.1%) | 0.269 | 0.433 | No |
| | Internet search | 104/217 (47.9%) | 97/206 (47.1%) | 0.922 | 0.974 | No |
| | Family or friends | 21/217 (9.7%) | 46/206 (22.3%) | <0.001 | 0.002 | Yes |
| | Previous personal experience | 57/217 (26.3%) | 49/206 (23.8%) | 0.576 | 0.738 | No |
| | Not verifying | 0/217 (0.0%) | 2/206 (1.0%) | 0.237 | 0.395 | No |
| | Other | 4/217 (1.8%) | 0/206 (0.0%) | 0.124 | 0.247 | No |
| Q35 | Never | 150/217 (69.1%) | 132/206 (64.1%) | 0.302 | 0.470 | No |
| | Rarely | 35/217 (16.1%) | 38/206 (18.4%) | 0.607 | 0.744 | No |
| | Sometimes | 20/217 (9.2%) | 28/206 (13.6%) | 0.170 | 0.312 | No |
| | Always | 0/217 (0.0%) | 1/206 (0.5%) | 0.487 | 0.656 | No |
| | Frequently | 12/217 (5.5%) | 7/206 (3.4%) | 0.351 | 0.515 | No |

– continues on the next page –

– continues from the previous page –

| Question | Response item | HB | NHB | Raw *p*-value | BH adjusted *p*-value | Significance |
|---|---|---|---|---|---|---|
| Q36 | Strongly disagree | 116/217 (53.5%) | 104/206 (50.5%) | 0.560 | 0.725 | No |
| | Disagree | 58/217 (26.7%) | 61/206 (29.6%) | 0.518 | 0.684 | No |
| | Neutral / neither disagree nor agree | 33/217 (15.2%) | 36/206 (17.5%) | 0.599 | 0.744 | No |
| | Agree | 7/217 (3.2%) | 5/206 (2.4%) | 0.772 | 0.864 | No |
| | Strongly agree | 3/217 (1.4%) | 0/206 (0.0%) | 0.249 | 0.407 | No |

** HB - Respondents with a healthcare background, NHB - Respondents without a healthcare background*
