# Supplementary material for: OTC Medication Risk Literacy, Confidence, and Safety Behaviours Among Romanian Adults: A Cross-Sectional Scenario-Based Assessment
Source: Healthcare (Basel). 2026 Sep 7;14(17):2886. doi: 10.3390/healthcare14172886 (PMC13565273; doi:10.3390/healthcare14172886)
Supplement: Supplementary file 1 [file healthcare-14-02886-s001.zip › healthcare-4495687-Supplementary_Table_S3.pdf]

Supplementary Table S3. Pairwise post hoc comparisons for scenario-based recognition of OTC medicine-related risks (Q10 – Q21) and self-assessed knowledge and confidence items (Q22-Q27) by healthcare status. Legend: HP – healthcare professionals, HS – healthcare students, NHP – respondents with non-healthcare background.

Question	HP vs HS	HP vs NHB	HS vs NHB
Q10	0.712	**0.015**	**0.004**
Q11	0.712	**<0.001**	**0.007**
Q12	0.854	0.089	0.274
Q13	0.712	0.092	**0.038**
Q14	0.582	**0.022**	**0.003**
Q15	0.269	**<0.001**	**<0.001**
Q16	1.000	0.091	0.091
Q17	1.000	**0.012**	**0.034**
Q18	0.483	**0.014**	**0.001**
Q19	1.000	0.106	0.091
Q20	1.000	0.716	0.712
Q21	1.000	0.582	0.712
Q22	0.382	**<0.001**	**0.004**
Q23	0.138	**<0.001**	**<0.001**
Q24	0.476	**<0.001**	**<0.001**
Q25	0.426	**0.008**	**0.037**
Q26	0.269	**<0.001**	**<0.001**
Q27	**0.018**	**<0.001**	**<0.001**
