# Supplementary material for: OTC Medication Risk Literacy, Confidence, and Safety Behaviours Among Romanian Adults: A Cross-Sectional Scenario-Based Assessment
Source: Healthcare (Basel). 2026 Sep 7;14(17):2886. doi: 10.3390/healthcare14172886 (PMC13565273; doi:10.3390/healthcare14172886)
Supplement: Supplementary file 1 [file healthcare-14-02886-s001.zip › healthcare-4495687-Supplementary_Table_S1.pdf]

Supplementary Table S1. Anthropometric and sociodemographic characteristics of the participants

Item	Variable	Category / statistic	n	%
Q1	Age	Minimum	18	-
		Mean	32.5	-
		Maximum	78	-
	Age group	18 - 24 y	171	40.4
		24 - 35 y	96	22.7
		35 - 50 y	100	23.6
		50 - 65 y	46	10.9
		> 65 y	10	2.4
Q2/Q3	BMI category	<18.5	29	6.9
		18.5 - 25	222	52.5
		25 - 30	95	22.5
		30 - 35	56	13.2
		35 - 40	15	3.5
		> 40	6	1.4
Q4	Gender	M	88	20.9
		F	334	79.1
Q5	Occupational status	Student	161	38.1
		Employed	225	53.2
		Unemployed	23	5.4
		Retired	14	3.3
Q6	Financial situation	I can afford most necessary things and some extra expenses	181	42.8
		I can comfortably cover basic needs	101	23.9
		I can cover basic needs, but have very little left over	87	20.6
		I have difficulty covering basic needs	23	5.4
		I prefer not to answer	31	7.3
Q7	Highest completed education	Primary school	13	3.1
		Secondary school	192	45.4
		College or university degree	129	30.5
		Master's degree / PhD	89	21.0
Q8	Respondent group	Healthcare background	217	51.3
		Non-healthcare background	206	48.7
	Healthcare professional role	Doctor	60	27.6
		Pharmacist	8	3.7
		Nurse / assistant	29	13.4
		Other healthcare professional	21	9.7
		Medical student	99	45.6
Q9	Residence	Urban	317	74.9
		Rural	106	25.1
